# Carbonic anhydrase inhibitors prevent presymptomatic capillary flow disturbances in a model of cerebral amyloidosis

**DOI:** 10.1002/alz.70023

**Published:** 2025-03-25

**Authors:** Eugenio Gutiérrez‐Jiménez, Peter Mondrup Rasmussen, Irene Klærke Mikkelsen, Sreekanth Kura, Signe K. Fruekilde, Brian Hansen, Luca Bordoni, Jasper Carlsen, Johan Palmfeldt, David A. Boas, Sava Sakadžić, Sergei Vinogradov, Mirna El Khatib, Jaime Ramos‐Cejudo, Boris Wied, Desiree Leduc‐Galindo, Elisa Canepa, Adam C. Mar, Begona Gamallo‐Lana, Silvia Fossati, Leif Østergaard

**Affiliations:** ^1^ Center of Functionally Integrative Neuroscience, Department of Clinical Medicine Aarhus University Aarhus Denmark; ^2^ Department of Biomedicine Aarhus University Aarhus Denmark; ^3^ Department of Biomedical Engineering Boston University Boston Massachusetts USA; ^4^ GliaLab and Letten Centre, Division of Anatomy Department of Molecular Medicine Institute of Basic Medical Sciences University of Oslo Oslo Norway; ^5^ Research Unit for Molecular Medicine (MMF), Department of Clinical Medicine Aarhus University Aarhus Denmark; ^6^ Athinoula A. Martinos Center for Biomedical Imaging Massachusetts General Hospital, Harvard Medical School Charlestown Massachusetts USA; ^7^ Department of Biochemistry and Biophysics Perelman School of Medicine University of Pennsylvania Philadelphia Pennsylvania USA; ^8^ Department of Chemistry School of Arts and Sciences University of Pennsylvania Philadelphia Pennsylvania USA; ^9^ Department of Psychiatry and Neurology New York University (NYU) Grossman School of Medicine New York City New York USA; ^10^ Alzheimer's Center at Temple Department of Neural Sciences Lewis Katz School of Medicine Temple University Philadelphia Pennsylvania USA; ^11^ Department of Neuroscience and Physiology Neuroscience Institute New York University (NYU) Grossman School of Medicine New York New York USA; ^12^ Section of Neuroradiology Department of Radiology Aarhus University Hospital Aarhus Denmark

**Keywords:** Alzheimer's disease, capillary dysfunction, capillary flow disturbances, capillary stalls, carbonic anhydrase inhibitors, presymptomatic

## Abstract

**INTRODUCTION:**

Disturbances in microvascular flow dynamics are hypothesized to precede the symptomatic phase of Alzheimer's disease (AD). However, evidence in presymptomatic AD remains elusive, underscoring the need for therapies targeting these early vascular changes.

**METHODS:**

We employed a multimodal approach, combining in vivo optical imaging, molecular techniques, and ex vivo magnetic resonance imaging, to investigate early capillary dysfunction in C57BL/6‐Tg(Thy1‐APPSwDutIowa)BWevn/Mmjax (Tg‐SwDI) mice without memory impairment. We also assessed the efficacy of carbonic anhydrase inhibitors (CAIs) in preventing capillary flow disturbances.

**RESULTS:**

Our study revealed capillary flow disturbances associated with alterations in capillary morphology, adhesion molecule expression, and amyloid beta (Aβ) load in 9‐ to 10‐month‐old Tg‐SwDI mice without memory impairment. CAI treatment ameliorated these capillary flow disturbances, enhanced oxygen availability, and reduced Aβ load.

**DISCUSSION:**

These findings underscore the importance of capillary flow disturbances as early biomarkers in presymptomatic AD and highlight the potential of CAIs for preserving vascular integrity in the early stages of AD.

**Highlights:**

- Uncovered early capillary dysfunction in a presymptomatic Alzheimer's disease (AD) mouse model.
- Evidence linking capillary stalls and capillary dysfunction with oxygen delivery issues in AD.
- Novel use of carbonic anhydrase inhibitors to prevent early capillary flow disturbances in AD.

## 1 BACKGROUND

Alzheimer's disease (AD) is the leading cause of dementia globally, affecting 46.8 million in 2015.^1^ In Europe, AD prevalence is predicted to double by 2050,^2^ underscoring the urgent need for effective disease‐modifying interventions. However, the precise biological causes of AD remain unclear. AD is understood as a biological and clinical continuum that develops gradually over years, marked by pathological changes such as amyloid beta (Aβ) plaques, cerebral amyloid angiopathy (CAA), hyperphosphorylated tau protein aggregation, and neurodegeneration.^3^

Supporting the continuum model of AD, human biomarker studies suggest that Aβ accumulation begins at least 15 years before clinical symptoms.^4^ Although therapeutic strategies targeting Aβ effectively reduce Aβ load, they have shown limited success in controlling disease progression and they fail to reverse memory loss.^5^, ^6^ This highlights the need to identify and target early disease mechanisms manifesting long before symptoms arise.

AD pathology and cerebrovascular disease (e.g., CAA) frequently coexist in older adults, with recent studies highlighting a potential synergistic relationship.^7^ Vascular contributions to AD are supported by changes in brain vasculature detected up to 10 years before symptomatic AD and possibly before Aβ deposits.^8^ AD also shares many risk factors with cardiovascular diseases.^9^, ^10^ Evidence from animal models shows vascular changes involving disturbed neurovascular coupling,^11^ blood–brain barrier (BBB) dysfunction,^12^ vascular oxidative stress, and inflammatory damage,^13^ ultimately reducing cerebral blood flow (CBF) and limiting oxygen and nutrient access to brain tissue. Regulating local CBF is essential for local oxygen delivery. Capillary transit time and blood flow distribution across the capillary bed significantly influence oxygen extraction.^14^ Disturbances in capillary flow dynamics including transient blockage by leukocytes or platelets, known as capillary stalls,^15^ and capillary constrictions due to Aβ plaques^16^ have been observed in animal models of AD. These disturbances may increase capillary resistance, disrupt flow distribution, and reduce oxygen extraction efficiency, termed capillary dysfunction.^17^

Recent observations highlight the importance of early capillary flow disturbances in AD pathology.^17^ Elevated capillary transit‐time heterogeneity (CTH) is observed in AD patients^18^ and individuals with mild cognitive impairment (MCI)^19^ and at preclinical stages.^20^ Similarly, a murine AD model exhibits increased CTH in symptomatic stages.^11^ However, capillary flow disturbances have not yet been observed during the presymptomatic stages of AD.

Therapeutic means to prevent the deterioration of capillary function may be crucial to prevent AD. Recent studies highlight carbonic anhydrase inhibitors (CAIs) as potential AD treatments.^21^, ^22^ CAIs are used clinically in treating glaucoma^23^ and altitude sickness.^24^ CAIs inhibit the reversible hydration of carbon dioxide (CO_2_) to bicarbonate and an hydrogen proton, a process playing a critical role in maintaining pH homeostasis.^25^ Elevated CO_2_ levels enhance CBF through vasodilation,^22^ whereas the resulting metabolic acidosis right‐shifts the hemoglobin–oxygen dissociation.^26^ Both of these actions therefore improve tissue oxygenation. Prior studies also show that CAIs reduce Aβ oligomer neurotoxicity by preventing mitochondrial dysfunction and pro‐apoptotic mechanisms.^21^, ^22^, ^27^ We recently showed that in 15‐month‐old C57BL/6‐Tg(Thy1‐APPSwDutIowa)BWevn/Mmjax (Tg‐SwDI) mice, long‐term CAI treatment with methazolamide (MTZ) or acetazolamide (ATZ) prevented cognitive decline, reduced vascular and total Aβ load, improved glial clearance, and limited inflammation.^28^ However, no studies have so far examined the effect of CAIs on brain microcirculation in vivo in mouse AD models.

This study aims to determine whether early capillary flow disturbances exist in presymptomatic Tg‐SwDI mice, a model of cerebral amyloidosis, and whether these alterations lead to capillary dysfunction (increased CTH), and impaired oxygen uptake. In addition, it examines whether early CAI treatment mitigates capillary flow disturbances in presymptomatic Tg‐SwDI mice, thereby improving oxygen availability, Aβ load, or brain structure.

Using a multimodal approach, we integrated in vivo optical imaging, molecular biology, and ex vivo magnetic resonance imaging (MRI) to investigate early capillary dysfunction in Tg‐SwDI mice and the impact of CAI treatment. The Tg‐SwDI model represents the AD continuum, with Aβ accumulation starting at 4 months without significant symptoms until 12 months.^29^ We demonstrate capillary hemodynamic disturbances in 9 to 10‐month‐old presymptomatic Tg‐SwDI mice. Early treatment, particularly with MTZ, significantly prevents these capillary flow disturbances, enhances oxygen transport, and prevents Aβ load. Our findings suggest that CAIs, particularly MTZ, may prevent vascular impairment and slow down AD pathology during the presymptomatic stages.

## 2 METHODS

### 2.1 Experimental model

The Danish Ministry of Justice and Animal Protection Committees approved all animal procedures and treatment, with the Danish Animal Experiments Inspectorate permit 2017‐15‐0201‐01241. We used adult homozygous mice from the Tg‐SwDI line (C57BL/6‐Tg(Thy1‐APPSwDutIowa)BWevn/Mmjax; MMRRC Stock No: 34843‐JAX, The Jackson Laboratory, Bar Harbor, ME, USA). A group of homozygous breeders was acquired from Jackson Laboratory and bred at the animal facility at the Biomedicine Department, Aarhus University. Animals were of both sexes. A group of control wild‐type (WT) mice (C57BL/6J) were acquired from The Jackson Laboratory, matching sex and age. After weaning from the breeder, mice were transported to the stable facility at the Center of Functionally Integrative Neuroscience (CFIN, Aarhus University). Mice were housed in group cages (between three and five mice/cage) with ad libitum access to water and a standard diet/treatment diet. Mice were maintained on a 12h:12 h light–dark cycle at constant temperature and humidity (21°C ± 2 temperature and mean ± SD; 45% ± 5 relative humidity). Behavioral studies were performed on mice with identical group characteristics, at the NYU School of Medicine Rodent Behavior Laboratory, and adhered to the guidelines of the NYU School of Medicine's Institutional Animal Care and Use Committee, as in previous work.^28^

**RESEARCH IN CONTEXT**

1. **Systematic review**: The existing literature demonstrates that microvascular flow disturbances are a characteristic of symptomatic Alzheimer's disease (AD). However, studies specifically examining these disturbances and therapeutic approaches in presymptomatic stages remain limited.
2. **Interpretation**: Our findings reveal that capillary flow disturbances and altered capillary morphology are present in the presymptomatic phase of AD in Tg‐SwDI mice, supporting the hypothesis that vascular dysfunction precedes cognitive symptoms. This study also expands on our previous work by being the first to demonstrate that carbonic anhydrase inhibitors (CAIs) can prevent these early vascular disturbances.
3. **Future directions**: This study underscores the need for further research into the mechanisms underlying early microvascular dysfunction in AD. In addition, this study supports the translational potential of CAIs for treatment of AD.

To assess sufficient statistical power, we conducted a power analysis based on previous work by Park et al., which demonstrated cerebrovascular disturbances in the Tg‐SwDI.^30^ Two reference age groups, 3 and 18 months, were used to estimate the cerebrovascular response metrics for the 9‐ to 10‐month cohort. The 9‐month sample data were calculated as the midpoint between the 3‐month data (WT: mean = 23 ± 1 standard error of the mean [SEM], Tg‐SwDI: mean = 16.5 ± 1 SEM) and the 18‐month data (WT: mean = 13 ± 1.5 SEM, Tg‐SwDI: mean = 9 ± 1.5 SEM) for the CBF response to whisker stimulation, as described in Figure 1A of Park et al. The initial power analysis indicated a required sample size of six mice per group to achieve 80% power with a significance level of .05. However, as our experimental design involves awake mice, where we anticipated a 60% success rate in completing experiments, we adjusted the sample size to account for potential losses. After this adjustment, the required sample size increased to 10 animals per sex per group.

**FIGURE 1:**
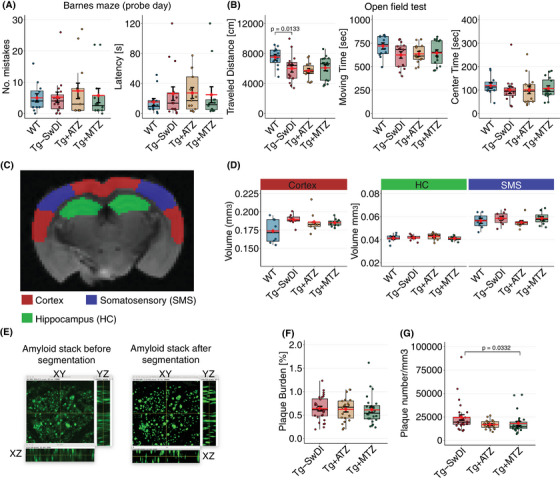
Early amyloidosis and cortical hypertrophy in cognitively normal Tg‐SwDI mice. (A) Analysis of the Barnes maze in WT and Tg‐SwDI mice with and without treatment. WT *n* = 12 [6 F, 6 M]; Tg‐SwDI *n* = 15 (5 F, 10 M); Tg+ATZ *n* = 13 (5 F, 8 M); Tg+MTZ *n* = 12 (6 F, 6 M). Plots represent the number of mistakes (left), and latency (right). (B) Analysis of open field to assess locomotor activity. WT *n* = 12 (6 F, 6 M); Tg‐SwDI, *n* = 15 (5 F, 10 M); Tg+ATZ, *n* = 14 (6 F, 8 M); Tg+MTZ, *n* = 16 (8 F, 8 M). Plots represent traveled distance (left), moving time (middle), and time spent on field's center (right). Traveled distance across groups (ANOVA: F‐value [3, 53] = 0.337, *p* = 0.798). WT versus Tg‐SwDI pairwise comparisons using EMMs, diff = –1493 ± 583 cm, t(53) = –2.561, *d* = 0.906. (C) Regional atrophy was assessed by estimating regional tissue volume using ex vivo MRI. Segmentation into cortex, hippocampus (HC), somatosensory area (SMS), and white matter (WM) was conducted. WT *n* = 8 (5 M, 3 F); Tg‐SwDI *n* = 9 (6 M, 3 F); Tg+ATZ *n* = 9 (4 M, 5 F); Tg+MTZ n = 12 (5 M, 7 F). (D) Plots for volume for each region. Cortex volume: WT versus Tg‐SwDI, F‐value [1, 15] = 6.629, *d* = 1.251. (E) TPM z‐stacks (100 slides per ROI; two ROIs per mouse) of methoxy‐positive amyloid deposits (green) were pre‐processed with ImageJ (left) and segmented using pixel classification of Ilastik (right). Tg‐SwDI, *n* = 28 ROIs in 15 mice (8 F, 6 M); Tg+ATZ, *n* = 21 ROIs in 15 mice (6 F, 8 M); Tg+MTZ, *n* = 24 ROIs in 12 mice (5 F, 7 M). (F) Plot of Aβ plaque burden (%). (G) Plot of plaque number per mm^3^. Tg‐SwDI versus Tg+MT, LMM, rank‐diff = –15.9 ± 6.31  cm, t(35.8) = –2.513, *d* = 0.957). Error bars = SD. Aβ, amyloid beta; ANOVA, Analysis of variance; ATZ, Acetazolamide; diff, difference, EMMs, estimated marginal means; F, female; LMM, linear mixed models; M, male; MTZ, Methazolamide, ROI, region‐of‐interest; Tg, transgenic; Tg‐SwDI, C57BL/6‐Tg(Thy1‐APPSwDutIowa)BWevn/Mmjax; WT, wild‐type.

### 2.2 Treatment randomization

For treatment with CAIs, a special diet was designed with either acetazolamide (ATZ; Santa Cruz Biotechnology, Inc. Dallas, Texas, USA) or methazolamide (MTZ; Santa Cruz Biotechnology, Inc.). Medication was added at a concentration of 0.01% (100 parts per million), corresponding to 20 mg/kg/day, to a standard diet (Altromin 1320; Brogaarden ApS, Lynge, DK). The three specialized diets were produced with the same pellet form. Researchers involved in the experimental acquisitions were blinded to the treatment. Treated animals were distributed randomly across three treatment groups, each with a specialized diet containing methazolamide (Tg+MTZ), acetazolamide (Tg+ATZ), or placebo (standard diet: Tg‐SwDI), while maintaining balance between female and male mice across groups. Because reports have shown that vascular impairments^30^ are already present in the Tg‐SwDI mice at the age of 3 months, we anticipated that starting the treatment at 4 months age would slow the vascular impairment in the treated groups. The treatment lasted 6 months, and the scans were performed when the mice were between 10 and 11 months of age.

### 2.3 Behavioral tests

During the dark phase of the light cycle, mice underwent a behavioral test battery and were acclimated to the testing room for at least 30 min prior to each session.

#### 2.3.1 Barnes maze

The Barnes maze assesses visuospatial learning and memory in mice. Our standardized Barnes maze protocol is based on numerous past reports using both bright light and a white‐noise stimulus to motivate escape from the open, brightly lit maze that is associated with aversive noise.^31^, ^32^ The test comprises a raised beige platform (91.5 cm in diameter), with a textured surface, positioned 91.5 cm above the ground, which features 20 evenly spaced holes around the edge, each 5 cm wide, located 2.5 cm from the perimeter. Underneath one of these holes, a gray plastic escape box (10 × 8.5 × 4 cm) was placed, and its position was kept consistent throughout trials. The placement of the target hole was varied among mice or groups to ensure balanced spatial conditions. Visual cues were positioned in the testing environment and remained constant across the testing period. Illumination was provided by an overhead lamp (600 lux at the surface of the maze) positioned above the center of the maze. Over seven consecutive days, mice underwent 12 trials: one habituation trial on Day 0, 10 training trials spanning days 1 to 5 (2 trials/day), and the final probe trial on Day 6. During the habituation phase, mice were introduced to the maze under an inverted, clear 500 mL beaker for 1 min, accompanied by white noise. The white noise was generated by a San Diego Instruments (San Diego, CA, USA) white noise generator located above the maze, with the sound level set at 75  dB measured at the maze surface. Subsequently, the beaker was slowly lifted to reveal the target hole, allowing the mouse to explore the escape box for 2 min. Following a 2 h intertrial interval (ITI) of habituation, standard training trials commenced, with each day featuring two trials; the animal was placed in the center of the maze in a plastic (15 × 15 × 20 cm) start box, with a white noise stimulus applied. After 10–15 s, the box was lifted and the animal was given a 3‐min window to locate and enter the designated escape hole. Upon successful entry, the white noise ceased, and the mouse was left in the escape box for 1 min, and then moved to the home cage. If the mouse failed to find the escape hole within the allotted time, it was gently guided to the target hole beneath the inverted beaker, and left there for 1 min, with white noise off. On each training day, trials were separated by 1.5 to 2 h ITIs, with the maze platform rotated in each trial. On the sixth day, a 2‐min probe trial was conducted, like the training trials, except that the escape box was removed. Behavior was recorded via an overhead camera for subsequent analysis (Noldus Ethovision XT 11.5 software, Noldus Worldwide, Wageningen, NL). Key metrics, including distance traveled and number of non‐target holes visited, were measured as indicators of spatial memory and then plotted accordingly.

#### 2.3.2 Open‐field test

The open‐field test was employed to evaluate animal locomotor activity patterns. The test occurred within a novel open‐topped white acrylic enclosure, measuring 40 × 40 × 40  cm, with uniform overhead lighting (110–130 lux). Mice were individually introduced into a corner of the arena and given 15 min to freely explore. Video of the 30‐min sessions was recorded from an overhead camera and subsequently analyzed (Noldus Ethovision XT 11.5 software, Noldus Worldwide). Locomotor activity parameters, such as total distance traveled, speed, moving time, and time spent in the central zone (20 × 20  cm) were recorded, and hence plotted.

### 2.4 Surgical preparation and training of animals for optical imaging in the awake state

A chronic cranial window was surgically implanted over the somatosensory area of the barrel cortex (S1bc), as described previously.^33^ To minimize stress and enhance recovery, mice were acclimated to handling for 5 days prior to surgical preparation. On the day of the surgery, mice received a subcutaneous injection of dexamethasone (4.8 mg/kg; Kalcex, Riga, Latvia) to reduce post‐surgical brain edema. The surgical procedure was performed under anesthesia with 1.75%–2% isoflurane (induction with 3%) with 100% O_2_ flow through a face mask. Subsequently, intraperitoneal injections of ampicillin (200 mg/kg) and carprofen (10 mg/kg) were administered for antibiotic and anti‐inflammatory/pain‐killer relief purposes, respectively. Lidocaine was applied under the scalp for local anesthesia and eye ointment was applied to both eyes. During the entire surgery, the mouse was securely mounted on a stereotaxic frame (RWD Life Science Co., LTD, Shenzhen, Guangdong, China). A cranial window of ≈3  mm in diameter was centered over the region of S1bc representing the whisker C2 (1.5–2 mm anterior‐posterior, 3 mm mediolateral). The cranial window was covered with an in‐house glued glass plug consisting of one 5‐mm and three 3‐mm pieces of cover glass. The glass plug was secured to the skull with the application of cyanoacrylate glue around the edges (Loctite Super Glue gel; Loctite Brand, Westlake, Ohio, USA). A custom‐made head bar was affixed and glued onto the frontal area of the skull, and the whole exposed area of the skull was covered with a layer of dental cement (Meliodent, Kulzer. Hanau, Germany). After surgery, mice recovered in a heated recovery chamber and were monitored closely until voluntary movement was observed. Subsequently, each mouse was moved back to its own housing box. We continued to monitor recovery for a period of 5 days post‐surgery, providing antibiotic and anti‐inflammatory medication as needed. Acclimatization with handling continued through the post‐surgical period to minimize stress and improve training sessions.

After the post‐surgical recovery period, mice underwent daily training sessions to acclimatize to awake imaging conditions, with training durations progressively increasing from 15 minutes to the target session length of ≈2.5 hrs. over a minimum of 10 days. This protocol was designed to minimize stress levels, as demonstrated by prior research from our group showing significant reductions in corticosterone levels and heart rate in mice after 10–11 days of such training, indicating stress habituation for both male and female mice.^34^ For training sessions, we used a replica of the custom‐built imaging frame to ensure familiarization with the restraint apparatus. After each session, mice were rewarded with condensed milk and returned to their cages. During the scanning session, mice were monitored through a night vision camera installed within the imaging systems. A rest day was provided between imaging sessions. The gradual habituation process implemented in our study was designed specifically to reduce stress‐induced vascular perturbations based on our previous experience,^34^, ^35^ thereby minimizing confounding effects on hemodynamic measurements. However, if hyperreactivity to handling or restraint was excessive, the scanning session was delayed after two additional days of training.

We placed a tail catheter for infusion of fluorescent dyes to label the plasma for two‐photon microscopy (TPM) imaging. For this procedure, mice were lightly anesthetized with isoflurane 1.2% delivered through a face mask (100% O_2_ flow). A catheter was assembled with a PE10 tube attached to a 50‐unit (500 µL) insulin syringe on one side and a 30G Monoject needle (Covidien Monoject, Cardinal Health, Dublin, OH, USA) on the opposite side. The catheter was filled with a 5% heparin solution (LEOPharma, Ballerup, Denmark). The catheter was placed into one of the lateral tail veins, after warming up the tail and applying ethanol to induce vasodilation. The catheter was fixed with dental cement (GC‐Fuji‐Plus, GC Corporation, Japan) to avoid movement or detachment during the scanning period. After the scanning session, the catheter was gently removed, and the tail was gently pressed until the bleeding stopped.

### 2.5 Optical imaging acquisition

We performed imaging in awake head‐restrained mice using a frame equipped with a head‐holder and a fabric cradle where the mouse could rest in a relaxed position.

For optical coherence tomography angiograms (OCT‐A), we applied a spectral‐domain OCT system (1310 nm center wavelength, bandwidth 1700 nm, Telesto II, Thorlabs, Inc.; Newton, New Jersey, USA). This system provides an axial resolution of ≈3.5 µm and an imaging speed of ≈47,000 A‐scan/s. To achieve a transverse resolution of ≈1.5 µm, we used a 10X near‐infrared (NIR) objective (Mitutoyo Corporation, Kawasaki, Kanagawa, Japan). Using the decorrelation method, we acquired time‐series OCT‐A, each consisting of 400 × 400 pixels (600 µm^2^).^36^ In our setup, each OCT‐A acquisition took ≈8.5 s with a buffering time of 5 s between OCT volumes. Sixty volumes of the cortical microvasculature were acquired in each region‐of‐interest (ROI).

#### 2.5.1 Detection of capillary stalls from post‐processed data

After postprocessing the OCT‐A, a volume of 30 pixels was selected 135–225 µm beneath the brain surface and was projected as maximum intensity projection (MIP) for analysis. In OCT imaging, stalling events are considered capillaries without perfusion during OCT‐A volume. OCT signal relies on the movement of particles and light path interference. Each volume represents 8.5 s of capillary dynamics. Stalled capillaries typically re‐perfuse within one or two volumes, although some stalls may last longer. We identified capillary stalling events using the graphical user interface (GUI) “CapStall” designed in MATLAB (MathWorks, Natick, Massachusetts, USA), which allowed a semiautomatic identification of the stall events.^37^ For postprocessing, two research assistants, blinded to the treatment randomization, inspected all images in a time series (volume scanned as a function of time) to identify all image frames in which the capillary segments stalled at least one time. After having done the identification of stalling capillaries, a stall‐o‐gram was plotted. Stall‐o‐grams depicted the capillary stalls and the stalling frequency during the 60 (1 volume = 8.5 s) volumes scanned. For statistical analysis, the assistants manually quantified the capillaries examined in each MIP using ImageJ and a MATLAB script to calculate the following parameters:

1. Total of identified capillary stalls.
2. Incidence rate, defined as the fraction of capillaries showing a stall episode for 8.5 min (sixty 8.5 s volumes).
3. Point of prevalence, which is the average number of capillary stalling episodes per volume (8.5 s).
4. Cumulative stall time, representing the cumulative fraction of stalling time relative to the total scanning time (≈8.5 min).

#### 2.5.2 Two‐photon imaging

All TPM imaging experiments were performed using a Bruker Ultima‐IV two‐photon laser scanning microscope (Brucker Corporation, Billerica, Massachusetts, USA) and PrairieView Software, version 5.1 (Brucker Corporation, Billerica, Massachusetts, USA). Excitation of fluorescent dyes was performed with a tunable Ti:Sapphire laser Chameleon (Coherent, Santa Clara, CA, USA). Fluorescence emission was detected by two GaAsP photomultiplier modules (Hamamatsu, H10770. Hamamatsu City, Japan). We used an Olympus 10x water immersion objective (0.3 numerical aperture [NA]; 3.3 working distance [WD]) to image 1.18 mm^2^ field of view (FOV) for bolus tracking, intravascular pO_2_ estimations, and reference angiograms for bolus tracking. We used an Olympus 20x water immersion objective (1.0 NA, 3.00 mm WD) for capillary linescans, high‐resolution z‐stacks for angiograms, and Aβ plaques. To avoid any effect of temperature on brain hemodynamics, we heated the objective lens with an electric heater (TC‐HLS‐05, Bioscience Tools, San Diego, CA, USA) to maintain the temperature of the water between the cranial window and the objective lens at 36°C–37°C.

Two imaging sessions were performed, interleaving 1 day between scans to reduce stress induced by repetitive sessions. All scans were performed during steady‐state conditions. On the first day of TPM imaging, we acquired measurements of brain hemodynamics in awake‐restrained mice. The examinations on Day 2 did not include data that are sensitive to movement and physiological variations (e.g., capillary diameter), and they were therefore performed under slight anesthesia (0.9%–1% Isoflurane) delivered through a face mask as we acquired morphological data to avoid excessive movement during the ≈10 min acquisition of each z‐stack (i.e., angiograms, Aβ plaques).

TPM acquisition sequences:

1. Indicator–dilution technique (Bolus tracking): We estimated mean transit time (MTT) and CTH applying an indicator–dilution methodology as described previously.^38^ This methodology relies on the detection of concentration–time curves (CTCs) during the passage of a bolus of fluorescent dye from the arterial to the venous network. Each bolus was injected through the tail catheter using an infusion pump (GenieTouch; KenScientific, Torrington, USA) set at an infusion rate of 20 µL/s. To avoid pain due to the intravenous infusion of cold solutions, we kept the catheter warm with a rubber tube attached to a standard heating circulation bath (ThermoScientific, Waltham, MA, USA). To locate the ROI, we identified the shadows of the pial vessels detecting reduced Nicotinamide Adenine Dinucleotide (NADH) autofluorescence (820 nm excitation). The autofluorescence signal was filtered with a 460 ± 25 nm bandpass filter (Chroma Technology, USA). The volume of the catheter was ≈60 µL and was filled with heparin solution before the scan; therefore, we habituated the mice to the bolus injection ≈6 times (10 µL boluses) before performing the recording paradigm to fill up the catheter with the fluorescent dye. We identified arterial and venous networks during the habituation paradigm. Each bolus consisted of 10 µL of the fluorescent dye Texas‐Red dextran (70,000 mW; Thermo Fischer Scientific). The signal was filtered with a bandpass filter centered at a wavelength of 605 nm with a bandwidth of ±35 nm (Chroma Technology Corp., Bellows Falls, VT, USA). We identified an ROI as an area where both pial arteries and veins converged, and a large region with capillaries was observed. Each bolus was infused after 7 s from the initiation of a time‐series of two‐dimensional (2D) spiral scans with a spatial resolution of 256 × 256 (pixel size = 2.30 µm), which yielded a frame rate of ≈6.98 frames per second (fps). A total of three boluses of each mouse were included for analysis.
2. Intravascular pO_2_ measurements: After the placement of the tail catheter, a bolus of ≈40–50 µL (10–15 µM final concentration in the blood plasma) of the phosphorescent probe PtP‐C343 (≈32,000 mW).^39^ Using the residual green fluorescence of the C343‐antenna fragments in PtP‐C343, we performed a raster scan to map the pial vessels and quantify their diameter. The fluorescent signal was detected with a PTM and passed through a 525 ± 25 nm bandpass filter. We used the PointScan mode of the TPM software to obtain single‐point measurements within the luminal side of the arterial and venous networks of the most superficial layers of the brain cortex. We aimed to include two points per vessel in the same vessel detected during the indicator–dilution scans. Upon excitation at a single point during 10 µs (820 nm, ≈10 mW average power with beam modulation off), the phosphorescence output was passed through a 680 ± 30 nm bandpass filter and collected during 290 µs by a PMT module (H10770PA‐50; Hamamatsu), as described previously.^40^ For O_2_ estimates, we averaged ≈15,000 phosphorescence decays per point. Quenching of the probe's phosphorescence is dependent on the O_2_ concentration. Therefore, the phosphorescence lifetimes in arteries are expected to be shorter than in veins. Two trials per mouse were performed.
3. Linescans for capillary scans: After the indicator–dilution scans, the remaining volume required to reach 200 µL was injected through the tail catheter to enhance contrast for capillary scans. Capillary scans were conducted using Linescan mode of the TPM software with a 20x featuring a 1.0 NA and WD of 2.0 mm (UMPLFLN 20x, Olympus, Shinjuku City, Japan). Digital zoom was set to 5x, resulting in an *XY* pixel size of 0.23 µm. Capillaries were randomly selected, with efforts made to remain within the same FOV as used for the indicator–dilution scans. Fifty to 20 capillaries were scanned per mouse through depths between 50 and 200 µm from the surface (*Z* = 0). The number of capillaries was chosen to balance the time constraints of the scanning session, which also included additional methodologies. Depth was selected based on a previous observation of low variability in cell flux and velocity within this range.^38^, ^41^ A scan path was assigned in one to five capillaries, once in the axial direction of the capillary lumen for red blood cell velocity (RBCv) estimation, and twice transversally to the lumen for an averaged cell flux and capillary diameter. Linescan period varied between 0.5 and 2 ms per line. Scans were performed under steady‐state conditions for 5 s.
4. z‐Stacks for Aβ detection and angiograms: For detection of Aβ plaques, the day before the scanning session, the mice were injected intraperitoneally with a dose of 2 mg/kg of methoxy‐X04 dye (Tocris Bioscience, Bristol, UK). In our setup, the excitation peak was at 790 nm, which was selected for the scanning sessions. The signal was filtered with a 460 ± 25 nm bandpass filter. For the detection of vessels, we labeled plasma with 200 µL of Texas‐Red dextran 70,000 mW (5 mg/mL, Thermo Fischer Scientific, Waltham, MA, USA). Excitation of Texas‐Red dextran was performed using a wavelength of 900 nm and emission was filtered with a 605 ± 35 bandpass filter.

#### 2.5.3 Post‐processing of the indicator–dilution techniques

MTT and CTH were estimated by modeling the tissue transport function (TF) through deconvolution following the injection of a dye bolus, as described previously.^38^ For the analysis of the 2D time‐series, we developed a dedicated MATLAB GUI that allowed the selection of vessels within the FOV. For modeling TF, it was crucial to select an arterial input function (AIF) and a venous output function (VOF). Initially, we identified pial arteries and veins as vessels positioned parallel to the cortex and without diving into the brain tissue. The main feeding AIF was selected as the artery with the largest diameter and the shortest time‐to‐peak (TTP) of fluorescence intensity. VOF was defined as the vein with the largest diameter and with the longest TTP. We identified the main arteriolar AIF as a vessel branching from an artery (10 < *d *< 30 µm), with the shortest TTP. The main venular VOF was defined as an ascending vessel (10 < *d* < 60 µm) with an anastomosis with a pial vein and with the shortest TTP. The vascular TF described the relative amount of dye emerging at the VOF as a function of time after an idealized, instantaneous bolus injection on the AIF. The TF was modeled by a gamma probability density function with parameters α and β, and a scaling factor, f. With this TF, we estimated MTT as the mean (α⋅β) and CTH as the standard deviation (SD) (√α⋅β). To visualize curve fitting and verify the MTT estimation, the AIF and VOF were compared after time‐shifting the VOF curve by the estimated MTT (Figure S1). Bad curve fitting or curve‐intensity saturation was used as a criterion for rejection of analyzed pairs (Figure S2).

#### 2.5.4 Post‐processing of lifetime imaging for pO_2_ measurements

To estimate pO_2_, we determined the phosphorescence lifetime by fitting the phosphorescence intensity decay with a single exponential function using the nonlinear least‐squares method. The lifetime was converted to pO_2_ using the calibration plot obtained in independent oxygen titration experiments.^39^ For statistical analysis, we averaged the two measurements per vessel. To correlate oxygen tension with the diameter of the pial vasculature, two research fellows, blinded to the treatment, performed segmentation and diameter estimation, averaging the distance of five transversal lines placed transversal to the vessel axis using the ROI manager plug‐in of FIJI.

#### 2.5.5 Post‐processing of single capillary scans

Before processing, all images were filtered using a top‐hat filter to improve signal‐to‐ noise ratio (SNR). RBCv, capillary cell flux, and diameter were calculated using a MATLAB GUI, as performed previously.^38^ Briefly, RBCv was calculated from the axial capillary line scan using the Radon transform algorithm for accurately and robustly analyzing streak angles.^42^ Calculations were performed in 150 ms windows, displaced by 50 ms for each iteration. Cell flux was calculated by analyzing the intensity variations from the cross‐section scan of each capillary. Average intensity profiles were derived from 150‐ms time interval windows within transversal line scans. A cluster analysis was applied to determine the presence of cells as they passed through the capillary. The capillary diameter was estimated from cross‐sectional scans as the full width at half‐maximum (FWHM) of intensity profiles within 150 ms windows,^43^ disregarding time intervals with the presence of RBCs.

#### 2.5.6 Post‐processing of angiograms for capillary morphometrics

The identification and segmentation of the capillary network involved the following steps:

1. Raw image stacks of the vasculature were first pre‐processed using a Macro written in ImageJ (National Institutes of Health, Bethesda, Maryland, USA) for contrast enhancement and image denoising. The macro involved the following steps:
  - CLIJ2_equalizeMeanIntensitiesOfSlices for bleaching correction.
  - Contrast enhancement with normalization (saturated pixels = 0.4%).
  - Background subtraction (rolling ball = 50).
  - 3D filtering (median 3D; x = 2, y = 2, z = 2).
2. Vessel segmentation was performed using DeepVess, a deep convolutional neural network that enables the identification of individual capillary segments by extracting the vessel center line.^44^
3. Identification of capillary segments after segmentation was performed using a dedicated MATLAB script described previously.^44^ Post‐processing included filtering according to diameter to remove the large vessels, with a set diameter limit of 8 pixels, equivalent to 9.2 µm (*XY* resolution = 1.15 µm per pixel). The vessel diameter and its SD were calculated for each segment by measuring the distances of all non‐zero voxels within the segment's binary skeleton to the nearest boundary of the vessel segmentation. The diameter was defined as twice the mean of these distances, whereas the SD was computed from the same distance measurements to capture the variability in vessel diameter along each segment.
4. Finally, another dedicated MATLAB script was employed to estimate capillary tortuosity, as the capillary segment length divided by the Euclidean distance between the segment endpoints.

Additional parameters estimated: (1) capillary diameter coefficient of variance (COV) as diameter SD along the segment divided by the mean diameter along the segment; (2) capillary length density as the total capillary length (µm) in the total z‐stack volume (µL); and (3) capillary blood volume as the fraction of the sum of capillary volumes (µL) and the total z‐stack volume (µL).

#### 2.5.7 Post‐processing for Aβ deposits quantification

Aβ quantification was performed using a combination of macros designed using ImageJ and Ilastik, an interactive machine learning for bioimage analysis.^45^

A sub‐stack of 100 slices (100 µm) was selected from a z‐stack scanned with TPM to maintain consistency between all groups. Contrast enhancement and denoising were performed using the same macro as described for the angiograms. Segmentation of plaques was conducted using the pixel classification workflow of Ilastik, which assigns labels based on pixel features and user annotations using a Random Forest classifier. The software was trained using two sub‐stacks, and the rest of the stacks were classified using the batch mode. The Ilastik model's performance was validated using the raw binary image as the ground truth, as it represents the amyloid signal in its entirety, free from noise and additional artifacts. The Ilastik model's performance was validated using the raw binary image as the ground truth, as it represents the amyloid signal in its entirety, free from noise and additional artifacts. The model's segmentation accuracy was assessed on 25‐slice and 100‐slice test sets to evaluate its effectiveness on three‐dimensional image stacks. For the 25‐slice test set, the model achieved a dice similarity coefficient (DSC) of 0.832, precision of 0.801, and recall of 0.866. On the 100‐slice test set, the model demonstrated improved performance, achieving a DSC of 0.843, precision of 0.766, and recall of 0.938. These results indicate robust segmentation performance, particularly in capturing true positives (high recall), and a consistent overlap between the predicted and ground truth segmentations (DSC). Finally, for quantification of Aβ deposits, we use the “3D Objects Counter” from ImageJ, which gave the volume of each plaque across the sub‐stack.

### 2.6 Extraction of tissue samples for molecular analyses

Following the last imaging session, mice underwent euthanasia by induction of deep anesthesia with isoflurane (4% in FiO_2_ 25%) and subsequent administration of an overdose of pentobarbital (100 mg/kg) intraperitoneally. Upon confirmation of respiratory arrest, a transcardial extraction of blood (300–500 µL) was performed. Plasma was recovered after centrifugation to remove the blood cells. Subsequently, the brain was carefully extracted from the skull of each mouse. The hippocampus and the region of the cortex of the scanned area were extracted (left hemisphere). All tissue samples were promptly snap‐frozen and stored at –80°C until further preparation for molecular analyses.

### 2.7 Enzyme‐linked immunosorbent assay (ELISA) to determine specific protein levels

The frozen tissue samples were finely minced on an aluminum plate cooled below 0°C, and further homogenized by grinding in a 1.5 mL LoBind (Eppendorf, Hamburg, Germany) tube with a plastic pestle (GE Healthcare, Sample Grinding Kit), in 20 µL per mg tissue of an extraction buffer with 2% sodium dodecyl sulfate (SDS), 100 mM sodium HEPES (4‐(2‐hydrosyethyl)‐1‐piperazineethanesulfonic acid), and 1 cOmplete Protease Inhibitor (PI) Tablet (Roche, Basel, Switzerland) per 10 mL (1x PI). Finally, the sample was ultrasonicated for three cycles of five ultrasonication pulses on a Branson Sonifier 250 (Branson Ultrasonics Corporation, Brookfield, CT, USA) at output control 3 and 30% duty cycle. The protein content of each sample was measured in triplicate with the Pierce bicinchoninic acid assay (BCA) Protein Assay Kit (Thermo Fisher Scientific) on a BSA standard curve according to the manufacturer's protocol.

Samples were diluted in extraction buffer to 2 µg protein/µL. To replace SDS with the ELISA‐compatible buffer Tween‐20, SDS was precipitated by the addition of an equal volume of a Tween/K buffer with 0.1% Tween‐20, 100 mM K_2_HPO_4_, 1 × PI buffer followed by centrifugation for 15 min at 15,000×*g* at 2°C, to pellet the potassium–SDS precipitate. The supernatant was aliquoted for each of the ELISAs, and total protein content was determined, in triplicate, with the BCA assay.

For use in the ELISA standard curves, a sample buffer was prepared by precipitating SDS from the extraction buffer with the Tween/K buffer as described.

All samples and standards were measured in duplicate. Absorbance was measured at 450 nm. The observed absorbance in the 0 standard was subtracted from all measurements. A four‐parametric standard curve was applied to calculate specific protein concentrations. The average of the duplicates was multiplied by the dilution factor and normalized to the total protein concentration of the sample after SDS removal. The results are reported as pg‐specific protein/mg total protein.

Aβ40 was detected by an ELISA kit (Termo Fisher Scientific, Cat. #: KHB3481, Waltham, MA, USA) targeting human Aβ40. Transgenic mice samples were diluted by a factor of 200 in the kit “Standard Diluent Buffer.” The Aβ40 standard was reconstituted in 55  mM sodium bicarbonate, pH 9.0, according to protocol, and diluted from 100 ng/mL to the range of the standard curve (7.81–500 pg/mL) in a 1:199 mix of sample buffer and kit “Standard Diluent Buffer.” The assay was otherwise run according to the manufacturer's protocol. The manufacturer reports a 0.5% cross‐reactivity with rodent Aβ40, which may explain the trace amount of signal observed in some samples from WT mice, which were furthermore diluted by only a factor of 5.

For the detection of Aβ42, we used an ELISA kit (Termo Fisher Scientific, Cat. #: KHB3441) targeting human Aβ42. Samples were diluted by a factor of 5 in the kit “Standard Diluent Buffer.” The Aβ42 standard was reconstituted and diluted in a 1:4 mix of sample buffer and kit “Standard Diluent Buffer.” The assay was run otherwise according to the manufacturer's protocol.

For detection of cyclophilin A (CypA), we used an ELISA kit (Abbexa, Cat. #: abx585050, Miltin, CA, UK) targeting murine CypA. Samples were diluted by a factor ca 1.3 in the kit “Standard Diluent Buffer” (80 µL sample + 25 µL kit buffer). The CypA standard was reconstituted and diluted in a 3.8:1.2 mix of sample buffer and kit “Standard Diluent Buffer.” Color development was allowed to proceed for 60 min. The assay was run otherwise according to the manufacturer's protocol.

Intercellular adhesion molecule 1 (ICAM‐1) was detected using an ELISA kit (Abcam, Cat. #: ab100688, CA, UK) targeting murine ICAM‐1. Samples were diluted by a factor of 3.6 in the kit “1x Assay Diluent B” (28.5 µL sample + 103.5 µL kit buffer). The ICAM‐1 standard was reconstituted and diluted in a 30:11.4 mix of sample buffer and kit “Standard Diluent Buffer.” The assay was run otherwise according to the manufacturer's protocol.

### 2.8 Mesoscale discovery

Quantification of vascular endothelial growth factor A (VEGF‐A) in plasma samples (1:1) obtained from scanned animals was conducted using the U‐Plex Mouse VEGF‐A Mesoscale Assay (Mesoscale Discovery, Rockville, USA) based on electrochemiluminescence. The procedure adhered to the standard protocol recommended by the manufacturer for preparation and measurements.

### 2.9 Ex vivo MRI

An independent sample of mice was used for ex vivo MRI scans. Four experimental groups were studied: WT mice (C57BL/6J, *N* = 8; F = 3, M = 5), Tg‐SwDI mice treated with MTZ (Tg+MTZ, *N* = 12; F = 7, M = 5), Tg‐SwDI mice treated with ATZ (Tg+ATZ, *N* = 9; F = 5, M = 4), and Tg‐SwDI mice treated with placebo (Tg‐SwDI, *N* = 9; F = 3, M = 5). Ex vivo MRI was chosen over imaging in anesthetized mice due to logistical constraints in monitoring the animals during extended scanning sessions.

Upon euthanasia, mice underwent perfusion–fixation with heparin (10 U/mL)–treated 0.9% saline (pH = 7.3) for 2 min, followed by ice‐cold 4% buffered paraformaldehyde (PFA, pH = 7.2–7.4) for 2 min. Subsequently, the brain was removed from the skull and placed in PFA for extended fixation. In preparation for imaging, samples were washed in phosphate‐buffered saline (PBS; Sigma USA, P4417‐ 50TAB) for a minimum of 24 h to improve the signal by removal of excess fixative. The sample was then securely placed in a magnetic resonance imaging (MRI)–suitable tube filled with fluorinert (FC‐770, 3M  inc., Saint Paul, MI, USA) as is standard.^46^

MRI was conducted using a Bruker Biospec 9.4T preclinical MRI system (Bruker Biospin, Ettlingen, Germany) equipped with a bore‐mounted 15 mm volume coil. High‐resolution structural data for volumetric estimation was acquired at 150 µm in‐plane resolution in 200‐µm‐thick slices using a segmented (eight segments) T2‐weighted echo planar imaging (EPI) sequence. Scan parameters were 16 averages, echo time (TE) = 31.7 ms, repetition time (TR) = 4500 ms, Bandwidth (BW) = 277 kHz, 70 axial slices. ROIs (i.e., cortex, somatosensory cortex [SMS], hippocampus [HC], and white matter [WM]) were manually segmented using the open software ITK‐SNAP (General Public License), as shown previously.^47^ The volume of each of these regions was then calculated as the number of voxels in each ROI multiplied by the nominal voxel volume (150 µm × 150 µm × 200  µm, i.e., 0.0045 mm^3^ per voxel).

### 2.10 Statistical analysis

We used R version 4.0.3 to perform statistical to assess our two hypotheses: (1) baseline model differences (WT vs Tg‐SwDI), and (2) treatment effectiveness, comparing the Tg+ATZ and Tg+MTZ groups relative to Tg‐SwDI. For behavioral analysis and MRI analyses, a two‐way analysis of variance (ANOVA) was performed to determine overall differences among the groups (WT, Tg‐SwDI, Tg+ATZ, Tg+MTZ) with sex (female, male) as a between‐mouse factor.

Parametric analysis was performed for normally distributed data (evaluated using Q–Q plots, Shapiro–Wilk tests, or the Kolmogorov–Smirnov test for larger datasets). For variables that did not meet normality assumptions, we applied log‐transformation and reassessed normality. If normality was achieved following log‐transformation, parametric analyses were performed as described later. For variables that remained non‐normal after log‐transformation and without repeated measurements per mouse, we conducted non‐parametric analyses using either the Kruskal–Wallis test and pairwise Wilcoxon tests with Benjamini–Hochberg correction.

In all analyses of optical imaging data, outliers were identified by calculating the z‐score of each variable and removing data with a z‐score below –3.29 or above 3.29, as described previously.^48^ We used the lme4 package for linear mixed‐effects model (LMM)^49^ estimates and constructed models with the hemodynamic parameter (e.g., cumulative stalling time, MTT, CTH) as the dependent parameter. As independent parameters, we included group category as a fixed effect and subjects as a random effect (e.g., *MTT ∼ group + (1 | mouse ID)*). For variables that did not meet normality assumptions, log‐transformed values were used after testing normality again. For variables that remained non‐normal after log‐transformation, we conducted LMM with ranked values for semi‐parametric analyses to account for the repeated measurements.

To examine the effect of gender, we further expanded the model to include gender as an additional fixed effect. These two models were compared using a likelihood ratio test using the function “anova” in R [e.g*., anova(model with sex effect, model without sex effect)*] to assess whether including sex as a fixed effect significantly improved the model fit. If a significant effect of sex was observed, the parameter estimates were adjusted for sex in subsequent analyses.

If the LMM encountered a singular fit, indicating that the random intercept for mouse did not add value, we simplified the model to a linear model (LM) without the random effects. Multiple comparison was performed using estimated marginal means (EMMs) with the “emmeans” package based on either the LMM or the simplified LM. EMMs were calculated for each group, and specific contrasts were defined to evaluate the baseline model differences and treatment effectiveness. Custom contrasts were applied with Benjamini‐Hochberg (BH) adjustment to control for multiple comparisons.

We estimated effect sizes by calculating Cohen's *d* ^50^ for parametric analysis reaching significance or trends (*p* ≥ 0.05 and < 1). Effect sizes were categorized as small (*d* = 0.2), medium (*d* = 0.5), and large (*d* ≥ 0.8) based on standard conventions. For non‐significant trends, power analysis to determine the sample size required to achieve 80% power (1‐β = 0.8) at a significance level of α = 0.05. This approach rigorously evaluates trends, providing insights into both their statistical significance and biological relevance.

To assess the relationship between MTT and CTH, we performed a correlation analysis across two parts of the vascular networks: artery‐to‐vein and arteriole‐to‐venule pairs: artery‐to‐vein and arteriole‐to‐venule pairs. This analysis utilized a linear model (LM) to determine the strength and direction of correlation within each group. The models were adjusted by the bolus trial to control for variability introduced by bolus injection characteristics.

All normality tests and the statistical test or models selected for each variable are shown in Supplementary Materials 1.

All plots were constructed using the “ggplot2” package, displaying individual data points along with error bars representing the standard error of the mean (SEM). The results in the tables are presented as mean ± SD.

## 3 RESULTS

### 3.1 Middle‐aged Tg‐SwDI mice are characterized by normal memory function, despite amyloidosis and motor function abnormalities

To confirm that 9‐ to 10‐month‐old Tg‐SwDI mice are a suitable model of presymptomatic AD, we compared spatial memory and locomotion to those of WT mice, using the Barnes maze. Notably, no significant changes in the number of errors or latency were observed between WT and Tg‐SwDI mice in the Barnes maze (Figure 1A), demonstrating preserved spatial memory function in the current settings. Our assessment was coupled to the open field test to assess locomotor activity, given that escape latency in the Barnes maze may be influenced by non‐cognitive factors such as motor function, motivation, anxiety, or general search strategy.^51^, ^52^ The test revealed motor changes in the Tg‐SwDI mice, characterized by a reduction in the distance traveled compared to WT, accompanied by a trend toward reduced moving time (ANOVA, *p* = 0.0654), with a moderate effect (Cohen's d = 0.746, Figure 1B). An estimated sample size of 30 mice per group was required to achieve statistical significance. These results demonstrate that in our settings, the changes in motor function did not influence the overall latency in the Barnes maze. This is consistent with recent studies that found no spatial learning and memory deficits in 9‐month‐old Tg‐SwDI mice.^28^, ^29^, ^53^

To detect structural, cortical, or hippocampal changes, we estimated regional tissue volumes by ex vivo MRI in a group of 9‐ to 11‐month‐old Tg‐SwDI and WT mice. Cortex, HC, SMS, and WM were segmented, providing volumes for each region (Figure 1C). Notably, Tg‐SwDI mice did not show signs of atrophy but rather a trend toward increased cortical volume compared to the WT (Kruskal–Wallis, *p* = 0.0833; Figure 1D). The observed trend corresponds to a large effect size (Cohen's d = 1.121). These results are consistent with previous observations of whole‐brain hypertrophy in another model of cerebral amyloidosis^54^ and observation in patients with AD.^55^

To detect Aβ burden, we used volumetric TPM scans and the Aβ fluorescent tracer, methoxy‐X04 (Figure 1E). We confirmed that 9‐ to –10‐month‐old Tg‐SwDI mice show extensive Aβ burden (Figure 1F,1G).

Overall, our analysis of the Tg‐SwDI strain in the 9‐ to 11‐month age range revealed no brain atrophy or no deficit in spatial learning and memory despite the presence of Aβ in the brain tissue, underscoring its suitability as a model for the presymptomatic period of the AD continuum.

### 3.2 Capillary flow disturbances are present in presymptomatic Tg‐SwDI mice

**FIGURE 2:**
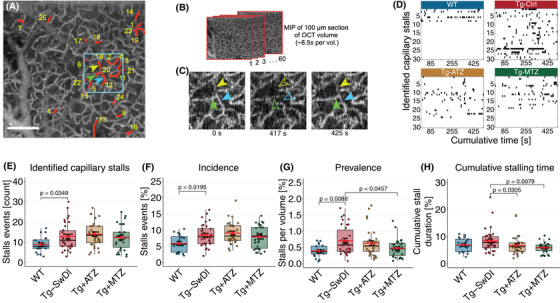
Long‐lasting capillary stalling in the Tg‐SwDI is prevented by CAIs. (A) OCT‐A was used to detect stalling events (ROIs per mouse). WT *n* = 20 ROIs in 11 mice (6 F, 5 M); Tg‐SwDI n = 34 ROIs in 18 mice (11 F, 7 M); Tg+ATZ *n* = 32 ROIs in 17 mice (7 F, 10 M); Tg+MTZ *n* = 30 ROIs in 15 mice (7 F, 8 M). MIP of 100 µm (axial) of an ROI (600 µm^2^) with stalling segments indicated (red markings). (B) Sixty OCT‐A volumes in two ROIs per mouse were performed (≈8.5 s/volume). (C) Magnification of selected region (cyan‐colored square in a) with capillary segments (arrowheads) with temporary perfusion interruptions. Hollow arrowheads indicate a stalling event. (D) Representative stall‐o‐grams from an ROI per group of stalling segments through 60 OCT‐A volumes. Black points represent stalling events per each image frame. (E) Identified capillary stall events in each examined ROI. WT versus Tg‐SwDI, LMM, log‐scale difference (log‐diff.) = 4 ± 1 stalls, t(63.1) = 2.398, *d* = 0.721.** (**F) Incidence of capillary stalls. WT versus Tg‐SwDI, LMM, diff. = 2.52 ± 1.05%, t(63.1) = 2.398, *d* = 1.002. (G) Prevalence as a percentage of stalling events per volume. For comparisons between Tg‐SwDI and WT group, a ranked LMM was used to account for non‐normality. WT versus Tg‐SwDI, LMM, rank‐diff. = 26.1 ± 9.63, t(60.4) = 2.707, *d* = 0.882; Tg‐SwDI versus Tg+MTZ, LMM, log‐diff. = –0.389 ± 0.167, t(58.1) = –2.338, *d* = 0.657. (H) Cumulative stall duration as percentage of time of each ROI (≈8.5 min). Tg‐SwDI versus Tg+ATZ, LMM, log‐diff. = –0.224 ± 0.102, t(116) = –2.190, *d* = 0.539; Tg‐SwDI versus Tg+MTZ, log‐diff. = –0.306 ± 0.104, t(116) = –2.941, *d* = 0.737. Scale bar = 100 µm. Error bars = SD. ATZ, acetazolamide; F, female; LMM, linear mixed model; M, male; MIP, maximum‐intensity‐projection; MTZ, methazolamide; OCT‐A, Optical coherence tomography angiogram; ROI, region‐of‐interest; SD, standard deviation; Tg, transgenic; Tg‐SwDI, C57BL/6‐Tg(Thy1‐APPSwDutIowa)BWevn/Mmjax; WT, wild‐type.

We examined presymptomatic Tg‐SwDI mice for capillary stalling, a phenomenon first described in AD models and linked to reduced CBF.^15^ Using OCT‐A (Figure 2A–D),^37^ we observed an increased number of capillary stalls in Tg‐SwDI mice compared to WT mice (Figure 2E and Table S1), including higher incidence (Figure 2F) and prevalence (Figure 2G). However, no significant difference in the cumulative stall duration was found between WT and Tg‐SwDI mice (Figure 2H). We did not observe a correlation between the incidence of stalling events and the cumulative stall duration (Figure S3B), indicating that the frequency of stalling events may not predict the total time capillaries remain stalled.

**FIGURE 3:**
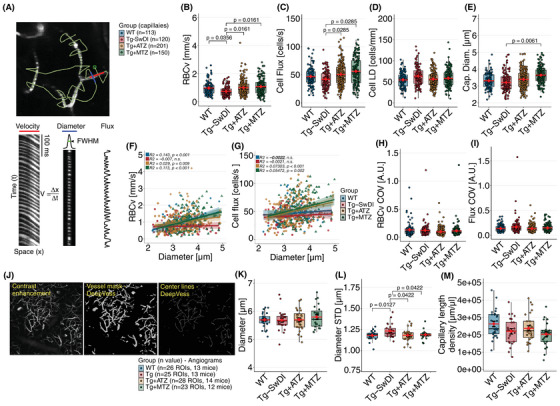
Disturbances in capillary flows and morphology are prevented in CAI‐treated Tg‐SwDI mice. (A) Simultaneous estimation of RBCv, cell flux, capillary LD, and capillary diameter (Cap. Diam.) from single‐capillary scans. A path traversed the capillary longitudinally (red) and transversely (blue) TPM line scans were performed (*upper left*). RBCv (*bottom left*) was calculated using the Radon transformation based on streak angles.^41^ Diameter (*bottom middle*) and cell flux (bottom *left*) were estimated from the transversal. A total of 583 capillaries were scanned across groups [WT, 6 mice (3 F, 3 M ); Tg‐SwDI, 7 mice (5 F, 2 M ); Tg+ATZ, *n* = 10 mice (5 F, 5 M); Tg+MTZ, *n* = 9 mice (6 F, 3 M )]. Scale bar: 20 µm. (B) Plot of the RBCv. WT versus Tg‐SwDI, LMM, log‐diff = –0.282 ± 0.128, t(31) = –2.197, *d* = 0.548. Tg‐SwDI versus treated groups, ranked LMM to account for non‐normality. Tg‐SwDI versus Tg+ATZ, rank‐diff = 92.2 ± 35.9, t(31.7) = 2.568, *d* = 0.608. Tg‐SwDI versus Tg+MTZ, rank‐diff = 94.5 ± 37.3, t(33.0) = 2.537, *d* = 0.624. (C) Plot of the capillary cell flux. For comparisons, a ranked LMM was used to account for non‐normality. Tg‐SwDI versus Tg+ATZ, rank‐diff = 72.9 ± 34.5, t(31.5) = 2.296, *d* = 0.515. Tg‐SwDI versus Tg+MTZ, rank‐diff = 91.4 ± 35.8, t(32.9) = 2.549, *d* = 0.594. (D) Plot for capillary linear density (cell flux/RBCv). (E) Plot of capillary diameter. Tg‐SwDI versus Tg+MTZ, LMM, log‐diff = 0.1087 ± 0.0337, t(29.9) = 3.226, *d* = 0.770. (F–G) Scatter plots illustrating the relationship between RBCv and capillary diameter (F) and cell flux and capillary diameter (G). Positive correlations in treated groups suggest improved capillary function. Plots of RBCv COV (H) and cell flux COV (I). (J) Postprocessing of TPM angiograms using DeepVess. Each angiogram was composed of 100 slices, ≈50 µm below the top layer. Each stack was pre‐processed for contrast enhancement (*left*) and then segmented into binary images and vessel center lines using DeepVess (*middle and right*)^44^ from where mean capillary segment diameter and standard deviation (SD) were estimated. Scale bar: 200 µm. Estimates were performed per mouse and per group. (K) Plots for mean capillary segment diameter. (L) Plots of mean capillary segment diameter SD. For comparisons a ranked LMM was used to account for non‐normality. WT versus Tg‐SwDI, LMM, rank‐diff = 22.8 ± 8.83, t(51.9) = 2.582, *d* = 0.950; Tg‐SwDI versus Tg+ATZ, LMM, rank‐diff = –20.4 ± 8.67, t(52) = –2.355, *d* = 0.851; Tg‐SwDI versus Tg+MTZ, LMM, rank‐diff =–18.9 ± 9.08, t(53) = –2.082, *d* = 0.788. (M) Plot of mean capillary length density. Error bars = SD. ATZ, acetazolamide; F, female; LD, linear density; LMM, linear mixed model; M, male; MTZ, methazolamide; RBCv, red blood cells velocities; SD, standard deviation; Tg, transgenic; TPM, two‐photon microscopy; Tg‐SwDI, C57BL/6‐Tg(Thy1‐APPSwDutIowa)BWevn/Mmjax.

We also examined single capillary hemodynamics using TPM (Figure 3A). We found lower RBCv (Figure 3B) and cell flux (Figure 3B and Table S2) in Tg‐SwDI mice compared to WT mice. Capillary linear density (flux/RBCv) and diameter were similar across the two groups (Figure 3D,3E), suggesting that capillary perfusion is maintained, consistent with previous observations in 12‐month‐old Tg‐SwDI mice.^56^ However, the reduced RBCv may indicate subtle impairments in flow regulation, potentially reflecting early capillary dysfunction or altered vascular tone in presymptomatic Tg‐SwDI mice.

In WT mice, RBCv showed a positive correlation with capillary diameter (Figure 3F), whereas cell flux did not (Figure 3G). This indicates that autoregulation in healthy mice, likely mediated by pericytes,^57^ ensures uniform red blood cell flux across capillaries, with velocity moderately scaling with diameter. In contrast, Tg‐SwDI mice exhibited no correlation between RBCv or flux and capillary diameter, suggesting that such autoregulation is disrupted. The absence of correlation is hence indicative of morphological or pathological factors in Tg‐SwDI mice that impede normal modulation of RBCv.

Finally, we calculated the COV of RBCv and cell flux, reflecting capillary flow distribution (Figure 3H, I). Although Tg‐SwDI mice exhibited a slightly higher cell flux COV compared to WT, this difference did not reach statistical significance, suggesting that capillary perfusion is maintained despite potential disturbances in capillary flow.

Collectively, these findings reveal early microvascular alterations in presymptomatic Tg‐SwDI mice, including increased capillary stalling, reduced RBCv, and disrupted flow regulation. These changes, although subtle, suggest that early capillary dysfunction may precede overt reductions in blood flow or oxygen delivery, potentially contributing to the progression of AD‐related pathology over time.

### 3.3 Disturbed capillary flow dynamics in presymptomatic Tg‐SwDI are associated with alterations in capillary morphology

To examine whether capillary flow changes relate to altered capillary morphology, we examined TPM angiograms (Figure 3J). The mean capillary diameter in Tg‐SwDI mice showed no significant difference compared to WT mice (Figure 3K and Table S3). However, Tg‐SwDI mice displayed increased diameter variability, as indicated by higher SD (Figure 3L) and COV along the capillary segments (Figure S4A and Table S3). This variability suggests that Tg‐SwDI mice may experience irregular capillary constrictions, possibly linked to pericyte activity, as observed in animal models^58^, and ex vivo studies.^16^ These morphological disruptions may provide a structural basis for impaired capillary flow.

We also examined capillary tortuosity, which was similar in Tg‐SwDI mice compared to WT mice (Figure S4B), aligning with observations in the APP/PS1 AD model.^44^ Notably, sex‐adjusted analysis revealed trends toward lower capillary length density (*p* = 0.0531, Cohen's *d* = 0.818; Figure 3M) and lower capillary blood volume (*p* = 0.0783, Cohen's *d* = 0.760; Figure S4C and Table S3) in Tg‐SwDI compared to WT mice. These findings suggest early structural changes that might contribute to flow abnormalities in the Tg‐SwDI model.

### 3.4 Disturbed capillary blood flow dynamics and morphology are associated with increased CTH in presymptomatic Tg‐SwDI mice

**FIGURE 4:**
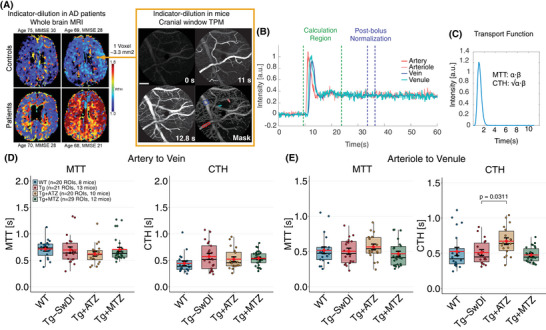
Capillary dysfunction is present in the Tg‐SwDI but not in the treated mice with CAIs. (A) Indicator–dilution method for estimating MTT and CTH in clinical settings using DSC‐MRI. Left: RTH maps (CTH/MTT) from two controls and two patients with different Mini‐Mental State Examination (MMSE) scores predicting AD patients (Modified from Eskildsen et al.^18^) Maps of MTT and CTH are estimated in each voxel through parametric deconvolution^60^ between an arterial input function (AIF, i.e., middle cerebral artery) and a residue function (signal residue after the passage of the contrast agent). In contrast, high‐spatial resolution of TPM (Right) allows the selection of AIF (red range colors) and a venous output function (VOF; blue range colors) within the field of view. Scans involved the injection of 10 µL of fluorescent dye while performing spiral scans (≈7 fps.). Scale bar: 100 µm. (B) Intensity curves of vessels selected. A 10 s curve‐matching region was selected to estimate MTT and CTH, and all curves were normalized to the post‐bolus baseline of the AIF. (C) The probability transport function of capillary transit times h(t) was parameterized by a gamma variate with parameters α and β (MTT = α·β, CTH = √α·β). (D) MTT and CTH estimations between arteries and veins. LMM were constructed to compare the effect of treatment in MTT, CTH. (E) MTT and CTH estimations between arterioles and venules. Tg‐SwDI versus Tg+ATZ log‐diff. = 0.261 ± 0.103, t(82) = 2.530, *d* = 0.846. Error bars = SD. AD, Alzheimer's disease; ATZ, acetazolamide; CAIs, carbonic anhydrase inhibitors; CTH, capillary transit‐time heterogeneity; DSC‐MRI, dynamic susceptibility contrast magnetic resonance imaging; fps, frames‐per‐second; LMM, linear mixed models; MTT, mean transit‐time; Tg, transgenic; RTH, relative transit‐time heterogeneity; TPM, two‐photon microscopy. SD, standard deviation; Tg‐SwDI, C57BL/6‐Tg(Thy1‐APPSwDutIowa)BWevn/Mmjax.

We measured MTT and CTH, with the latter serving as an index of capillary dysfunction,^59^ using indicator–dilution analysis developed for MRI in humans (Figure 4A)^60^ and adopted for TPM (Figure 4A–C).^38^ MTT and CTH were measured between two parts of the microvasculature: one from a pial artery to a vein, representing the entire vascular network of the scanned region and best at capturing the vasculature detected within a human MRI voxel, and one from an arteriole to a venule, reflecting the hemodynamics of a smaller subset of capillaries (Figure 4A).

A trend toward increased CTH was observed between arteries and veins in Tg‐SwDI mice (LMM, *p* = 0.0572), with a large effect size (Cohen's *d* = 0.9424), suggesting early‐stage capillary dysfunction. A power test calculation estimated a sample size of 19 mice per group required to achieve statistical significance. Although this trend fell short of conventional statistical significance, the effect size and alignment with elevated CTH in presymptomatic AD patients^20^ highlight its potential biological relevance. No differences in MTT were observed (Figure 4D, Table S4). No significant differences were observed in the MTT and CTH estimates between arterioles and venules when comparing WT and Tg‐SwDI mice (Figure 4E).

In passive compliant microvascular networks, MTT and CTH typically change in parallel as CBF increases, ensuring efficient oxygen extraction fraction (OEF), even at short capillary transit times.^61^ However, at low CBF (long MTT), active homogenization of capillary flows is believed to cause a deviation from this relationship,^62^ to ensure efficient oxygen extraction despite prolonged transit times. In our study, WT animals exhibited a low slope in the MTT–CTH relationship (Figure S5A), which we ascribe to this protective homogenization mechanism. In contrast, Tg‐SwDI mice showed a lack of this compensatory mechanism, with higher CTH values at longer MTT, indicative of impaired microvascular flow regulation.

### 3.5 Disturbed capillary flow dynamics in presymptomatic Tg‐SwDI mice do not affect oxygen extraction fraction (OEF)

**FIGURE 5:**
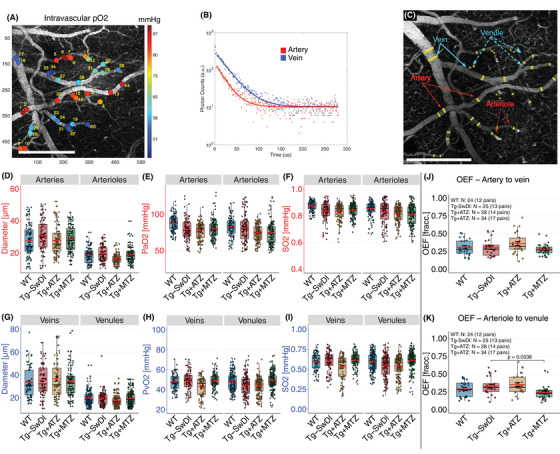
Alterations in OEF are not present in pre‐symptomatic Tg‐SwDI mice. (A) intravascular, pO_2_ was estimated using 2PLM by TPM after the I.V. injection of an oxygen‐sensitive dye (PtP‐C343). Single‐point excitation was used, and photons were collected with a PMT and counted using time‐correlated single‐photon counting (TCSPC). Two single‐point estimates were performed on each vessel from which MTT and CTH were estimated. (B) pO_2_ was determined by fitting phosphorescence intensity decay with a single‐exponential function using nonlinear least‐squares method. Lifetime was converted to pO_2_ using a calibration plot from independent oxygen titration. Higher photon lifetime in arteries indicates higher O_2_ concentration compared to veins. (C) Manual vessel diameter segmentation of vessels where pO_2_ was measured (mean of 5 lines per point; *N* value per vessels type and group in Table S5). Estimates from arterial networks (arteries and diving arterioles): Diameter (D), pO_2_ (E), and SO_2_ (F). WT versus Tg‐SwDI arterial SO_2_, LMM, rank‐diff. = –152 ± 75.5, t(57.1) = –2.017, *d* = 0.818. Estimates from venous networks (veins and venules): Diameter (G), pO_2_ (I), and SO_2_ (J). (K) OEF between pial arteries and veins (mean per mouse, two scans per mouse). (L) OEF between arterioles and venules. Tg‐SwDI versus Tg+MTZ, LMM, log‐diff. = –0.331 ± 0.134, t(57.2) = –2.461, *d* = 3.151. Error bars = SD. 2PLM, two‐photon phosphorescence lifetime imaging; I.V., intravenous; LMM, linear mixed model; MTZ, methazolamide; OEF, oxygen extraction fraction; PMT, photomultiplier; pO_2_, oxygen pressure; SD, standard deviation; Tg, transgenic; TPM, two‐photon microscopy; Tg‐SwDI, C57BL/6‐Tg(Thy1‐APPSwDutIowa)BWevn/Mmjax.

To evaluate the impact of capillary flow disturbances on OEF, we measured in vivo intravascular partial oxygen tension (pO_2_) using the oxygen‐sensitive dye PtP‐C343 with TPM phosphorescence lifetime (2PLM) as described previously^39^, ^40^ (Figure 5A,5B). Manual diameter estimations of scanned pial vessels at the region of 2PLM scans and segmentation of arteries, diving arterioles, upstream venules, and draining veins (Figure 5C) revealed no discernible diameter differences between Tg‐SwDI and WT (Figure 5D and Table S5).

We observed lower pO_2_ in the arteries and veins of male Tg‐SwDI mice compared to females, and statistical models were thus adjusted by sex. Tg‐SwDI mice exhibited trends toward lower pO_2_ (LMM, *p* = 0.0575; Figure 5E) and oxygen saturation (SO_2_, LMM, *p* = 0.0570; Figure 5F and Table S6), supported by large effect sizes (pO_2_ Cohen's *d* = 0.873, SO_2_ Cohen's *d* = 0.872). A sample size of 22 mice per group is estimated to achieve statistical significance. These trends suggest potential systemic factors influencing cerebral oxygenation, such as compromised myocardial function, observed in patients with AD.^63^

In WT mice, arterial pO_2_ increased with arterial diameter, explaining 15% of the variability in pO_2_ and SO_2_ across the arterial network (Figure S6A,B), consistent with previous studies in young WT (3‐month‐old).^64^ However, this correlation was absent in Tg‐SwDI mice, implying that other compensatory mechanisms, such as blood pressure regulation, may be influencing pO_2_ and SO_2_ levels in these mice. This observation aligns with reduced oxygen tension in brain parenchyma, a key regulator of oxygen diffusion,^64^ reported previously in an AD mouse model.^65^ No significant differences were found between WT and Tg‐SwDI mice in the diameter, pO_2_, and SO_2_ of veins or venules (Figure 5G–I).

Based on our pO_2_ measurements, we estimated the OEF by pairing the mean pO_2_ of arteries and veins (Figure 5J), and arterioles and venules (Figure 5K), for each mouse. We observed higher OEF in male mice than in female mice. After adjusting for the effect of sex, no significant differences in the averaged OEF per mouse were observed between WT and Tg‐SwDI mice, or between arteries and veins, or arterioles and venules.

### 3.6 Capillary stalling is associated with a higher expression of leukocyte adhesion molecules but not with BBB damage in presymptomatic Tg‐SwDI mice

**FIGURE 6:**
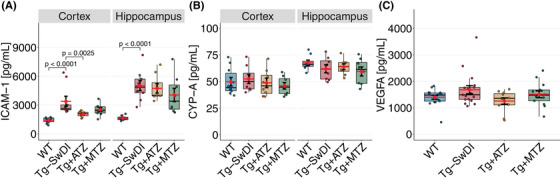
Leukocyte adhesion molecules and BBB marker levels. (A) Box plot of ICAM‐1 levels in homogenized cortex and hippocampus, respectively, measured by ELISA. Significant difference between groups in ICAM‐1 expression was observed in cortex (ANOVA, F‐value[1, 35] = 10.046, *p* < 0.001) and hippocampus (F‐value[1, 3] = 9.2224, *p* = 0.0019). Post hoc pairwise comparisons using estimated marginal means for cortex: WT versus Tg‐SwDI log‐diff. = 0.822 ± 0.114, t(35) = 7.206, *d* = 2.083; Tg‐SwDI versus Tg+ATZ log‐diff. = –0.429 ± 0.122, t(35) = 3.515, *d* = 1.086. Post hoc for hippocampus: WT versus Tg‐SwDI diff. = 3369 ± 701 pg/mL, t(29) = 4.803, *d* = 1.727. (B) Box plot of CYP‐A levels obtained from ELISA analyses of homogenized cortex and hippocampus. *N*‐value tissue ELISAs: WT: *n* = 12 (5 F, 7 M); Tg‐SwDI: *n* = 9 mice (5 F, 4 M); Tg+ATZ: *n* = 9 mice [5 F, 4 M; Tg+MTZ: *n* = 9 mice (5 F, 4 M)]. (C) Box plot of levels VEGF‐A in plasma measured by Mesoscale. N‐value tissue Mesoscale: WT: *n* = 17 (7 F, 10 M); Tg‐SwDI: *n* = 9 mice (10 F, 7 M); Tg+ATZ: *n* = 11 mice [7 F, 4 M; Tg+MTZ: *n* = 13 mice (5 F, 8 M)]. Error bars = SD. ANOVA, analysis of variance; ATZ, acetazolamide; BBB, blood‐brain barrier; CYP‐A, cyclophilin A; ELISA, enzyme‐linked immunosorbent assay; F, female; ICAM‐1, intracellular adhesion molecule 1; M, male; MTZ, methazolamide; SD, standard deviation; Tg, transgenic; Tg‐SwDI, C57BL/6‐Tg(Thy1‐APPSwDutIowa)BWevn/Mmjax; VEGF‐A, vascular endothelial growth factor A; WT, wild‐type.

Previous studies have linked AD models and capillary stalling with increased expression of adhesion molecules like ICAM‐1,^15^ indicative of endothelial activation. Our results show elevated ICAM‐1 levels in Tg‐SwDI compared to WT, confirming endothelial activation (Figure 6A). We also measured CypA molecular signature, linked to microvascular damage, including BBB breakdown,^66^ and assessed plasmatic levels of VEGF‐A, an essential pro‐angiogenic factor associated with capillary stalling.^67^ In contrast to the increase in ICAM‐1, tissue CypA and plasmatic VEGF‐A levels remained similar between groups (Figure 6B, C).

### 3.7 Effects of CAI treatment on cognitive performance, locomotion, and brain cortex volume in Tg‐SwDI mice

We administered CAIs, ATZ (Tg+ATZ), and MTZ (Tg+MTZ), to separate groups of Tg‐SwDI mice through the diet from 4 months of age. Both CAIs were provided at a dosage of 20 mg/kg/day through the diet (100 ppm). Spatial reference memory, assessed with the Barnes maze, showed no discernible effects of treatment at 9–10 months, noting that Tg‐SwDI and WT animals showed similar memory functions at this time (Figure 1A). Treatment with CAIs did not improve locomotor impairment compared to non‐treated Tg‐SwDI mice (Figure 1B).

Notably, neither treated groups showed any difference in brain cortex volume compared to the Tg‐SwDI mice (Figure 1D).

### 3.8 CAIs prevent capillary flow disturbances in presymptomatic Tg‐SwDI mice

MTZ and ATZ show reduced cumulative duration of capillaries affected by stalling compared to untreated Tg‐SwDI mice (Figure 2H), with MTZ‐treated mice also showing reduced stalling prevalence (Figure 2G). In addition, MTZ treatment led to significantly fewer prolonged capillary stalling events compared to Tg‐SwDI controls (Kolmogorov–Smirnov test: *p* = 0.002, Figure S3C), suggesting a more localized and pronounced effect than ATZ in mitigating capillary stalling.

Both treatment groups showed enhanced single capillary hemodynamics, as evidence by increased RBCv and cell flux compared to untreated Tg‐SwDI mice (Figure 3B, C). In addition, MTZ‐treated mice showed larger capillary diameters (Figure 3E), indicating enhanced capillary perfusion and reduced resistance. These findings suggest that CAI treatment alleviate capillary flow disturbances, potentially through improved capillary morphology or endothelial function.

Moreover, both treated groups show a proportional relationship between RBCv and capillary diameter, resembling the autoregulation observed in WT mice (Figure 3F). This relationship suggests that CAIs may have a protective effect on capillary flow regulation, disrupted in non‐treated mice. Enhanced cell flux in treated groups (Figure 3G) underscores how increased capillary diameter improves perfusion, with MTZ‐treated mice demonstrating a stronger effect. These findings underscore the therapeutic potential of CAIs, particularly MTZ, in preserving capillary flow regulation of AD models.

### 3.9 CAIs prevent abnormalities in the capillary morphology in presymptomatic Tg‐SwDI mice

Neither MTZ nor ATZ treatment significantly altered capillary diameter (Figure 3K). However, both reduced variability in diameter, as shown by decreased diameter SD (Figure 3L) and COV (Figure S4A and Table 3). These reduced values suggest fewer narrowing regions along the capillary lumen, potentially reflecting improved capillary integrity.

The reduction in diameter variability in CAI‐treated groups suggests fewer constricted capillary segments and improved pericyte regulation, although the modest changes raise questions about their attribution to treatment. Based on estimations from Nortley et al.^16^ (capillary diameter changes near pericytes) and Korte et al. (baseline diameters in AD), we calculated SDs of 0.182 µm (WT) and 0.207 µm (Tg‐SwDI), reflecting a 13.73% relative increase in diameter variability in Tg‐SwDI mice. Assuming pericytes influence 40 µm of their total 300 µm coverage,^68^ variance was scaled proportionally. CAI treatment reduced variability modestly: 4.31% for Tg+ATZ (0.051 µm) and 3.8% for Tg+MTZ (0.045 µm), but changes remained modest compared to WT (Supplementary Methods 1). These results suggest that CAIs partially improve pericyte regulation but do not fully reverse amyloid‐induced capillary dysfunction, likely due to limited pericyte influence and amyloid‐driven capillary tone changes beyond direct regulation.^16^, ^58^

Overall, these findings indicate that CAIs may help preserve capillary morphology in Tg‐SwDI mice by reducing diameter variability along capillary segments. This reduction in variability could signify fewer constrictions within the capillary lumen, potentially linked to improved pericyte function.

### 3.10 MTZ treatment prevents capillary dysfunction and improves oxygen availability in presymptomatic Tg‐SwDI mice

Treatment with CAIs showed differential effect on capillary flow distributions. Although no significant differences were observed in MTT across groups (Figure 4D,E, and Table S4), ATZ‐treated mice exhibited a significant increase in CTH at the arteriole‐to‐venule network (Figure 4E), suggesting less stable capillary flow regulation in response to perfusion changes in the local capillary network. Conversely, MTZ‐treated mice displayed reduced MTT‐CTH correlation at the artery‐to‐vein, resembling the active flow regulation observed in WT mice (Figure S5). This active regulation supports consistent capillary transit times under variable flow conditions, contrasting the passive regulation observed in Tg‐SwDI mice and partially in ATZ‐treated mice (*p* = 0.0753).

In addition, preventing impaired capillary flow regulation, MTZ‐treated mice showed lower OEF than untreated Tg‐SwDI between arterioles and venules (Figure 5J,5K, and Table S6). Despite reduction in capillary density (Figure 3M), indictive of lower surface area for oxygen exchange, the increased RBCv and cell flux (Figure 3C) and enlarged capillary diameters (Figure 3E) in MTZ‐treated mice likely enhance oxygen delivery efficiency. These changes, combined with shorter capillary stalls (Figure 2H), suggest that MTZ treatment alleviated the negative effects of reduced capillary length density, ensuring adequate oxygen availability during steady‐state conditions.

Altogether, these findings indicate that MTZ prevents capillary dysfunction and optimizes oxygen transport by enhancing flow dynamics, morphology, and delivery efficiency in presymptomatic Tg‐SwDI mice.

### 3.11 CAIs reduce ICAM‐1 expression and Aβ load in presymptomatic Tg‐SwDI mice

ATZ‐treated mice showed a significant reduction in ICAM‐1 expression in the cortex compared to non‐treated Tg‐SwDI mice, whereas MTZ‐treated mice exhibited a trend toward reduced ICAM‐1 expression (*p* = 0.0525, Cohen's *d* = 0.6205), suggesting a weaker effect relative to ATZ (Figure 6A). Both treatments exhibited no significant effects on CypA or VEGF‐A expression (Figure 6B,C).

In our previous study, CAI treatment prevented Aβ deposition in 15‐month‐old Tg‐SwDI mice.^28^ TPM measurements using methoxy‐X04 revealed a reduction of Aβ plaques in both treated groups. However, this reduction was statistically significant only in MTZ‐treated mice, whereas ATZ‐treated mice exhibited a trend toward reduction (*p* = 0.0717, Cohen's *d* = 0.730; Figure 1G). No significant changes were observed in total Aβ burden across groups (Figure 1F). Post hoc analyses revealed a reduction in small plaques (Figure S7A) and a trend toward fewer very small (*p* = 0.0675, Tg+ATZ Cohen's *d* = 0.155, Tg+MTZ Cohen's *d* = 0.843, Figure S7A) and medium‐sized plaques (*p* = 0.0677, Tg+ATZ Cohen's *d* = 0.6895, Tg+MTZ Cohen's *d* = 0.951, Figure S7C) in both groups. No difference in large plaques was observed (Figure S7D).

**FIGURE 7:**
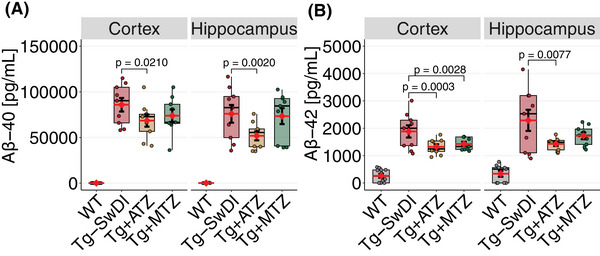
Amyloid overload is reduced in cortex and hippocampus in CAI‐treated Tg‐SwDI mice. (A) Box plot of total Aβ40 load measured by ELISA of homogenized cortex and hippocampus, respectively. Significant difference between groups was observed in log‐transformed Aβ40 load in cortex (F‐value[1, 2] = 4.0646) and hippocampus (F‐value[1, 2] = 3.14e9 pg/mL, *p* = 0.0011). Post hoc pairwise comparisons using estimated marginal means for cortex: Tg‐SwDI versus Tg‐ATZ diff. = –17416 ± 6253, t(23) = –2.785, *d* = 1.177. Pairwise comparisons for hippocampus: Tg‐SwDI versus Tg‐ATZ log‐diff. = –0.343 ± 0.091, t(23) = –3.762, d = 1.394. (B) Box plot of total Aβ42 load in cortex and hippocampus. Significant difference between groups was observed in log‐transformed Aβ42 load in cortex (F‐value[1, 2] = 11.173, *p* < 0.001) and hippocampus (F‐value[1, 2] = 6.9141 pg/mL, *p* = 0.0044). Post hoc pairwise comparisons using estimated marginal means for cortex: Tg‐SwDI versus Tg+ATZ log‐diff. = –0.317 ± 0.069, t(23) = –4.567, *d* = 1.598; Tg‐SwDI versus Tg+MTZ log‐diff. = –0.232 ± 0.069, t(23) = –3.341, *d* = 1.169. Pairwise comparison for hippocampus: Tg‐SwDI versus Tg+ATZ log‐diff. = –0.351 ± 0.109, t(23) = –3.214, *d* = 1.312. *N*‐value for cortex and hippocampus: WT: *n* = 12 (5 F, 7 M); Tg‐SwDI: *n* = 9 mice (5 F, 4 M); Tg+ATZ: n = 9 mice (5 F, 4 M; Tg+MTZ: *n* = 9 mice (5 F, 4 M). Error bars = SD. Aβ40, amyloid beta 40; Aβ42, amyloid beta 42; ATZ, acetazolamide; CAI, carbonic anhydrase inhibitor; ELISA, enzyme‐linked immunosorbent assay; F, female; M, male; MTZ, methazolamide; SD, standard deviation; Tg, transgenic; Tg‐SwDI, C57BL/6‐Tg(Thy1‐APPSwDutIowa)BWevn/Mmjax.

To further address the treatments’ impact on Aβ load, we quantified total Aβ40 and Aβ42 by conducting ELISA of homogenized cortex and hippocampus. Our analysis revealed lower levels of Aβ40 and Aβ42 load in the cortex and hippocampus of female mice. With sex‐adjusted analysis, mice treated with ATZ exhibited a reduced Aβ40 load in the cortex compared to non‐treated mice and in the hippocampus relative to Tg‐SwDI (Figure 7A and Table S7). MTZ‐treated mice also showed tendency toward a lower Aβ40 load in the cortex compared to Tg‐SwDI (*p* = 0.0673), with a large effect (Cohen's *d* = 0.811). Both treatment groups displayed a lower Aβ42 load in the cortex (Figure 7B and Table S7). However, only the Tg+ATZ group showed a significant decreased in Aβ42 load in the hippocampus (Figure 7B).

## 4 DISCUSSION

This study examined microvascular dynamics, capillary morphology, and Aβ load in presymptomatic, 9 to 10‐month‐old Tg‐SwDI mice with cerebral amyloidosis. Our first finding reveals that early microvascular changes, including capillary stalling, increased CTH, and capillary morphometric alterations, coexist with Aβ accumulation before cognitive impairment. These changes linked to endothelial activation may reduce oxygen delivery as the model ages. Our second main finding is that long‐term CAI treatment prevents microvascular disturbances, so treated mice had capillary morphometry similar to that of WT mice, reduced Aβ load, and fewer and shorter stalling events, possibly due to reduced endothelial activation.

Presymptomatic stages were defined by no memory impairment in the Barnes maze testing. Discrepancies with prior studies in Tg‐SwDI mice^69^, ^70^, ^71^, ^72^, ^73^ may result from methodological differences, such as maze design (8‐ vs 20‐hole maze) and outcome measures. Most deficits have been observed in studies in which only trial latency has been reported. However, escape latency might be impacted by differences in factors independent of cognition such as decreases in motor function, motivation, anxiety, or general search strategy.^51^, ^52^ In our study, escape latency did not differ between groups, suggesting that motor function did not affect performance. However, the Barnes maze alone may not fully capture subtle cognitive changes. Broader cognitive testing and larger cohorts are needed to confirm findings and evaluate vascular contributions to early AD stages in the Tg‐SwDI model.

Studies have demonstrated that capillary flow disturbances appear in AD models during advanced disease stages,^65^, ^67^, ^74^ and recent evidence shows that large vessel morphological changes and CBF reductions precede memory impairment in the 3xTg‐AD model.^75^ Our study extends these findings, showing that that capillary flow disturbances—marked by altered RBCv, cell flux, and capillary stalling—are present in presymptomatic mice with cerebral amyloidosis. This supports the notion that capillary dysfunction may precede CBF reductions, similar to AD patients during the prodromal phases.^19^, ^76^ Longitudinal studies addressing capillary flow disturbances at stages of the disease are necessary to understand the progression of capillary flow disturbances during the development of AD.

Capillary flow disturbances in the Tg‐SwDI mice are marked by increased CTH without changes in MTT, indicating impaired flow regulation despite preserved CBF. This dysfunction reflects disrupted oxygen extraction due to heterogenous capillary flow distribution.^59^ In WT mice, active flow homogenization stabilizes CTH during hypoperfusion, ensuring consistent oxygen delivery across capillaries as previously predicted.^14^ This mechanism, observed in prior studies with rats during hypovolemia^62^ and supported by our findings (Figure S5A), is absent in Tg‐SwDI mice. Instead, Tg‐SwDI mice exhibit passive flow regulation, as shown by the linear relationship between CTH and MTT.^61^ This lack of active adjustments heightens the brain's vulnerability to oxygen deficits during CBF reductions. These findings highlight the need to study CTH and MTT together to understand capillary dysfunction in AD.

Capillary dysfunction in AD likely involves multiple factors, including hematocrit, capillary diameter, length, and vessel stiffness.^77^ In WT and treated groups, RBCv and cell flux correlate with capillary diameter, highlighting the role of diameter in maintaining effective perfusion.^78^ However, this correlation is absent in Tg‐SwDI mice, suggesting structural or functional alterations in capillary walls. Evidence of pericyte constriction^16^ and dysfunction^79^ in AD supports the hypothesis that flow disturbances result from a combination of capillary stalls and pericyte rigidity or impairment.

**FIGURE 8:**
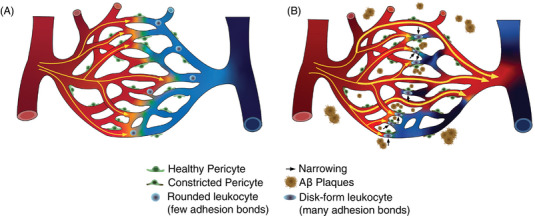
Disturbances in capillary patterns in Tg‐SwDI induce capillary dysfunction. (A) In healthy capillary networks, the transport of oxygen between blood and tissue is limited by the blood's capillary transit time and the distribution of capillary flows (thin yellow arrows) at a given time. Leukocytes commonly have a rounded shape with few adhesion bonds not affecting homogenization of capillary flows. (B) Phenomena affecting capillary patency, such as lumen narrowing and stalling, can disturb capillary flow patterns. In AD, narrowing is suggested to occur at regions at the pericyte soma.^16^ Consequently, leukocytes will need to squeeze through the capillaries, taking on a disk shape and increasing the number of adhesion bonds, which promotes capillary stalling. These pathological phenomena induce shunting of capillary flows through low‐resistance capillaries (thick yellow paths/arrows) and prevent their normal homogenization (increased CTH), leading to capillary dysfunction during increases in blood flow. AD, Alzheimer's disease; CTH, capillary transit‐time heterogeneity; Tg, transgenic; Tg‐SwDI, C57BL/6‐Tg(Thy1‐APPSwDutIowa)BWevn/Mmjax.

Morphometric data show increased variability in capillary diameter in Tg‐SwDI mice, indicating narrowed segments that may disrupt flow and increase resistance (Figure 8). These narrowed regions restrict leukocyte passage, forcing them to deform into disk‐like shapes and increasing their contact to the capillary wall as previously modeled.^80^ This elevated contact surface likely promotes the expression of adhesion molecules like ICAM‐1, as observed in our study. Increased ICAM‐1 expression exacerbates leukocyte adhesion, thereby compounding resistance and further impairing capillary perfusion. Figure 8 illustrates how these structural changes contribute to capillary dysfunction by diverting blood flow and reducing perfusion efficiency. Collectively these findings highlight capillary narrowing as a key driver of flow disturbances in presymptomatic Tg‐SwDI mice.

Reductions in pO_2_ and SO_2_ in pial arteries suggest factors influencing cerebral oxygenation, from local to systemic origin. Locally, this may involve the contribution of large vessels, which account for ≈50% of extracted O_2_ during steady‐state.^64^ Tissue pO₂ measurements in AD mouse models have demonstrated a reduction in tissue pO₂ compared to WT mice, accompanied by an increase in OEF.^81^ The elevated OEF reflects a compensatory response to impaired capillary flow regulation, aiming to preserve oxygen delivery despite vascular dysfunction. However, this compensatory mechanism can lead to decreased arterial SO₂ levels within the microvasculature and tissue, ultimately reflecting impaired oxygenation in AD brains.

Systemic factors, such as cardiac function or gas exchange impairments in the lungs, may also contribute to these changes in intravascular oxygen levels. Cardiac dysfunction, including Aβ deposition in the myocardium, has been reported previously in AD patients^63^ and the Tg2576 AD mouse model,^82^ underscoring the role of brain‐heart interactions in AD. In addition, respiratory impairment has been linked to damage in the brainstem, where nuclei involved in breath control are located.^83^, ^84^ Although evidence of disturbances in basal ventilation in AD animal models is scarce, respiratory impairment has been observed in the Tau‐P301L mouse model of tauopathy.^85^ These changes may not directly cause mortality; however, they can contribute to hypoxia and reduce SO₂ levels locally. Together, these findings emphasize the synergistic impact of systemic and local impairments, where cardiac and capillary dysfunction amplify each other's effects on brain perfusion. Further studies assessing tissue and intravascular oxygen levels, along with evaluations of cardiac and respiratory function, are needed to fully understand the mechanisms underlying reductions in pO₂ and SO₂ at the pial vasculature.

We demonstrate that CAIs prevent the development capillary flow disturbances, as evidenced by shorter capillary stalls, increased RBCv and cell flux, and reduced variability in capillary diameter in treated Tg‐SwDI mice, with no difference in diameter of pial vasculature. MTZ treatment further showed larger capillary diameters, reduced OEF, and decreased capillary density, suggesting improved oxygen delivery that may limit hypoxia‐driven angiogenesis. The lower OEF in MTZ‐treated mice likely reflects increased CBF and reduced CTH, enhancing oxygen delivery efficiency, as predicted by Jespersen and Østergaard^14^ and supported by indicator–dilution methods.^38^ In healthy vascular networks, such as in WT mice, reductions in CTH compensate for increased MTT, maintaining efficient oxygen extraction despite reduced CBF.^14^ MTZ‐treated mice showed similar adjustments of CTH whenever MTT increases, unlike non‐treated Tg‐SwDI and ATZ‐treated mice, indicating minimal active regulation. These findings suggest that MTZ preserved capillary flow regulation and oxygen availability, mitigating the effect of capillary dysfunction in Tg‐SwDI.

Previous studies have examined vasodilator treatments in AD models, such as nicorandil, which targets nitrate content and potassium channel activation, but these showed no improvement in CBF in the 3xTg AD model.^75^ Unlike nicorandil, CAIs demonstrate benefits that extend beyond vasodilation. They prevent mitochondrial dysfunction, inhibit pro‐apoptotic pathways, reduce glial reactivity markers, and activate anti‐inflammatory microglia,^21^, ^27^, ^28^ creating a healthier brain environment that promotes efficient Aβ degradation. These effects align with the reduced Aβ load observed in this and prior research.^28^ Further, evidence suggests that MTZ directly activates angiotensin‐converting enzyme II (ACE2), producing angiotensin (1‐7), a potent vasodilator associated with Mas receptor activation.^86^ This mechanism may enhance vascular tone and perfusion, contributing to the protective effects of MTZ. CAIs may also directly influence pericyte survival; inhibition of mitochondrial carbonic anhydrases (CA‐VA and CA‐VB) has been shown to prevent glucose‐mediated pericyte apoptosis in diabetic models.^87^, ^88^ Elevated CA‐VB levels in the Tg‐SwDI^28^ suggest that CAIs could counteract amyloid‐driven capillary dysfunction by supporting pericyte health. With our current animal model, we could not visualize pericytes, which prevented us from addressing their precise role in these mechanisms. Employing transgenic AD models with fluorescently labeled pericytes, as used in recent studies,^79^ could allow precise examination of pericyte‐mediated diameter changes and further evaluate their contribution to capillary dysfunction and the therapeutic efficacy of CAIs. Even without this information, our findings position CAIs as promising therapeutic candidates for mitigating capillary flow disturbances and broader cerebrovascular pathologies in AD.

Notably, we observed that treatment with ATZ showed an ambivalent effect, preventing Aβ load but with limited improvement in capillary hemodynamics. This discrepancy may be attributed to the known secondary effects of ATZ, such as its association with impaired diaphragmatic muscle function and reduced exercise capacity,^89^ and an increased risk of renal failure.^90^ Such adverse health effects might negatively impact vascular integrity and functional outcomes. MTZ, on the other hand, demonstrated a more robust impact on both capillary hemodynamics and Aβ pathology, potentially due to its additional mechanisms, such as ACE2 activation, which may amplify its vasoprotective effects. The divergence in the efficacy of these two CAIs highlights the importance of considering individual drug properties and off‐target effects in the development of therapeutic strategies for AD.

### 4.1 Study limitations

The temporal resolution of OCT‐A imaging (8.5 s per volume) may miss brief capillary stalls under one frame, potentially underestimating transient events. Conversely, including all stalls regardless of duration could inflate the representation of very short no‐flow events. Despite these limitations, our uniform and unbiased analysis across experimental groups ensured valid comparisons. Future studies integrating higher temporal resolution techniques or complementary flow measurements could further refine these observations.

Other limitations include a modest sample size and the absence of tau pathology. Future research with larger cohorts and tau‐inclusive models is needed to enhance findings.

Although systemic CAIs like ATZ and MTZ are used widely, side effects limit long‐term use,^24^ warranting the development of selective CA inhibitors and optimized dosing. To address these limitations, developing more selective CA inhibitors targeting specific enzymes or mechanisms, and optimizing dosing regimens to minimize side effects, may be necessary strategies for clinical applications^21^, ^91^, ^92^ (see also U.S. Patent No. 10780094).

## 5 CONCLUSION

Our findings highlight capillary flow disturbances and dysfunction as an early biomarker and therapeutic target in AD. Long‐term CAI treatment, particularly MTZ, prevents capillary dysfunction, brain remodeling, and amyloid load, positioning CAIs as promising treatments to slow AD progression.

## AUTHOR CONTRIBUTIONS

Eugenio Gutiérrez‐Jiménez, Silvia Fossati, and Leif Østergaard conceived the study. Eugenio Gutiérrez‐Jiménez established the study objectives, designed and performed the experiments, acquired and processed the TPM data, performed the statistical analysis, and wrote the first draft of the manuscript. Peter Mondrup Rasmussen, Irene Klærke Mikkelsen, and Sava Sakadžić set up and optimized TPM imaging processing software, and supported establishing TPM sequences and interpretation of data analysis. Furthermore, Peter Mondrup Rasmussen supported statistical analysis. Sreekanth Kura and David A. Boas supported with the OCT acquisition software and imaging post‐processing. Signe Fruekilde supported training of mice and scanning sessions. Brian Hansen performed, analyzed, and interpreted MRI imaging. Luca Bordoni established awake imaging in the laboratory and monitoring software together with Eugenio Gutiérrez‐Jiménez and Signe Fruekilde. Mirna El Khatib and Sergei Vinogradov synthesized and calibrated the oxygen probe PtP‐C343. Jasper Carlsen and Johan Palmfeldt performed molecular biology tests, data analysis, and interpretation. Jaime Ramos‐Cejudo reviewed the data analysis, manuscript, and supported the interpretation of the data. Boris Wied and Desiree Leduc‐Galindo performed the analysis of OCT data. Elisa Canepa, Adam C. Mar, and Begona Gamallo‐Lana performed behavior tests and processing of behavior data. Silvia Fossati contributed to the study objectives, experimental design, and interpretation of data, and critically reviewed the manuscript. Leif Østergaard mentored Eugenio Gutiérrez‐Jiménez, and contributed to the study design, objectives, and interpretation of data, and critically evaluated the manuscript.

## CONFLICT OF INTEREST STATEMENT

Silvia Fossati is an inventor on USA Patent No. 10780094 for the use of carbonic anhydrase inhibitors in Alzheimer's disease and cerebral amyloid angiopathy (CAA). All other authors have nothing to disclose in relation to this study. The other authors declare no conflicts of interest. Author disclosures are available in the Supporting Information.

## Supporting information



Supporting Information

Supporting Information

Supporting Information

Supporting Information

## Data Availability

Data for each plot and associated scripts: https://zenodo.org/uploads/14285647. Macros for preprocessing angiograms and stacks for amyloid quantification, and DeepVess postprocessing scripts: https://zenodo.org/records/13762334. Segmentation model for amyloid plaques: https://zenodo.org/records/14285562. Software utilized in prior studies is available upon request. If additional information or clarification regarding any tool or method is needed, we would be pleased to provide it.
