# Supplementary material for: Carbonic anhydrase inhibitors prevent presymptomatic capillary flow disturbances in a model of cerebral amyloidosis
Source: Alzheimers Dement. 2025 Mar 25;21(3):e70023. doi: 10.1002/alz.70023 (PMC11936728; doi:10.1002/alz.70023)
Supplement: Supplementary file 2 — Supporting Information [file ALZ-21-e70023-s002.docx]

Carbonic anhydrase inhibitors prevent presymptomatic capillary flow disturbances in a model of cerebral amyloidosis

Supplmentary material 1

Normality tests and statistical method selected for each variable.

1) Test for normality of WT vs. Tg-SwDI comparison

| Variable | Normality  WT vs. Tg-SwDI  Shapiro-Wilk test p-value | Log-normal  Shapiro-Wilk test p-value | Test selected |
| --- | --- | --- | --- |
| Barnes Maze |  |  |  |
| Errors | 3.716566e-05 | 0.2987084 | ANOVA-Log |
| Latency | 5.860845e-06 | 0.8303688 | ANOVA-Log |
| Open Field |  |  |  |
| Distance | 0.6494246 |  | ANOVA |
| Moving Time | 0.05547674 |  | ANOVA |
| Center Time | 0.001060286 | 0.04052316 | Kruskal-Wallis |
| Ex-Vivo MRI |  |  |  |
| Cortex | 0.01827955 | 0.01088643 | Kruskal-Wallis |
| SMS | 0.6969364 |  | ANOVA |
| Hippocampus | 0.5627711 |  | ANOVA |
| Stalls (2 ROIs per mouse | | | |
| Cap. No. | 0.2333365 |  | LM |
| Frequency | 0.0009953131 | 0.7682005 | LMM-Log |
| Incidence | 0.2193154 |  | LMM |
| Prevalence | 1.575693e-05 | 0.02094609 | LMM-Rank |
| Cumulative Time | 3.860248e-06 | 0.6196737 | LMM-Log |
| LineScans (Single capillary scans) | | | |
| RBCv | 3.813839e-09 | 0.3150592 | LMM-Log |
| Cell Flux | 2.673754e-07 | 1.21916e-05 | LMM-Rank |
| Diameter | 126501e-05 | 0.07250356 | LMM-Log |
| RBCv COV | 3.789611e-24 | 4.954588e-05 | LMM-Rank |
| Cell Flux COV | 8.303029e-25 | 0.0002861458 | LMM-Rank |
| Linear Density | 0.01103603 | 5.199404e-05 | LMM-Rank |
| Capillary morphometrics (Angiograms) | | | |
| Diameter | 0.08212172 |  | LMM |
| Diameter SD | 0.002272405 | 0.005093304 | LMM-Rank |
| Diameter COV | 0.6992053 |  | LMM |
| Capillary length density | 0.6532734 |  | LMM |
| Capillary Blood Volume | 0.206512 |  | LMM |
| Tortuosity | 5.709063e-07 | 2.712752e-06 | LMM-Rank |
| Indicator-Dilution (MTT and CTH) | | | |
| Artery-to-Vein | | | |
| MTT | 0.02899899 | 0.7198272 | LMM-Log |
| CTH | 0.0004584694 | 0.09315167 | LMM-Log |
| Arteriole-to-venule | | | |
| MTT | 0.02111958 | 0.9043851 | LMM-Log |
| CTH | 0.0001762859 | 0.02932177 | LMM-Rank |
| PO2 measurements | | | |
| Arteries | | | |
| Diameter | 0.004309591 | 1.565385e-06 | LMM-Rank |
| PO_2_ | 0.5579412 |  | LMM |
| SO_2_ | 2.541649e-11 | 1.905201e-13 | LMM-Rank |
| Veins | | | |
| Diameter | 2.543786e-20 | 1.244465e-05 | LMM-Rank |
| PO_2_ | 0.3808191 |  | LMM |
| SO_2_ | 0.0004000721 | 4.523638e-08 | LMM-Rank |
| Arterioles | | | |
| Diameter | 0.003179319 | 0.00027283 | LMM-Rank |
| PO_2_ | 0.006939339 | 0.005403462 | LMM-Rank |
| SO_2_ | 1.04513e-10 | 1.845004e-12 | LMM-Rank |
| Venules | | | |
| Diameter | 1.634185e-14 | 0.009639761 | LMM-Rank |
| PO_2_ | 0.5519759 | 0.0008332076 | LMM-Rank |
| SO_2_ | 1.198623e-05 | 3.95091e-13 | LMM-Rank |
| OEF | | | |
| OEF artery-to-vein | 0.009064316 | 0.1587285 | LMM-Log |
| OEF arteriole-to-venule | 0.3222819 | 0.1396941 | LMM-Log |

LMM: Linear mixed model; LMM-Log: Linear mixed model with log transformed values (natural log); LMM-rank: Linear mixed model with ranked values; LM: Linear model; ANOVA: Analysis of variance; ANOVA-Log: Analysis of variance with log transformed values (natural log).

2) Test for normality of Tg-SwDI vs. Tg+ATZ/Tg+MTZ comparison

| Variable | Normality  Tg-SwDI vs. Tg+ATZ/Tg+MTZ  Shapiro-Wilk test p-value | Log-normal  Shapiro-Wilk test p-value | Test selected |
| --- | --- | --- | --- |
| Barnes Maze | | | |
| Errors | 2.014323e-07 | 0.02346288 | Kruskal-Wallis |
| Latency | 5.33257e-07 | 0.2244373 | ANOVA |
| Open Field | | | |
| Distance | 0.2157412 |  | ANOVA |
| Moving Time | 0.0196188 | 0.01083023 | Kruskal-Wallis |
| Center Time | 0.0004023492 | 0.7105113 | ANOVA - Log |
| Ex-vivo MRI | | | |
| Cortex | 0.01389321 | 0.04679253 | Kruskal-Wallis |
| SMS | 0.1503364 |  | ANOVA |
| Hippocampus | 0.7611712 |  | ANOVA |
| Stalls (2 ROIs per mouse) | | | |
| Cap. No. | 5.849509e-05 | 0.03390529 | LM-Rank |
| Frequency | 0.007864594 | 0.01425508 | LMM-Rank |
| Incidence | 0.1329504 |  | LMM |
| Prevalence | 1.311057e-07 | 0.1108905 | LMM-Log |
| Cumulative Time | 1.399129e-09 | 0.2434813 | LMM-Log |
| LineScans (Single capillary scans) | | | |
| RBCv | 7.372803e-16 | 3.258449e-06 | LMM-Rank |
| Cell Flux | 0.0007717773 | 2.666392e-07 | LMM-Rank |
| Diameter | 4.902649e-05 | 0.05225171 | LMM-Log |
| RBCv COV | 6.801912e-35 | 8.033178e-09 | LMM-Rank |
| Cell Flux COV | 4.867385e-32 | 2.176049e-07 | LMM-Rank |
| Linear Density | 1.004029e-41 | 1.011506e-06 | LMM-Rank |
| Capillary morphometrics (Angiograms) | | | |
| Diameter | 0.2561872 |  | LMM |
| Diameter SD | 0.003759052 | 0.008190669 | LMM-Rank |
| Diameter COV | 0.4831506 |  | LMM |
| Capillary length density | 0.4082386 |  | LMM |
| Capillary Blood Volume | 0.1134689 |  | LMM |
| Tortuosity | 4.300921e-08 | 3.682106e-07 | LMM-Rank |
| Indicator-Dilution (MTT and CTH) | | | |
| Artery-to-Vein | | | |
| MTT | 9.061798e-07 | 0.006929009 | LMM-Rank |
| CTH | 0.009769759 | 0.7236355 | LMM-Log |
| Arteriole-to-venule | | | |
| MTT | 0.008556712 | 0.05836754 | LMM-Log |
| CTH | 0.0003454755 | 0.06462922 | LMM-Log |
| PO2 measurements | | | |
| Arteries | | | |
| Diameter | 0.0008880499 | 1.736167e-05 | LMM-Rank |
| PO_2_ | 3.038873e-05 | 0.001376317 | LMM-Rank |
| SO_2_ | 7.118201e-12 | 2.409352e-14 | LMM-Rank |
| Veins | | | |
| Diameter | 1.047229e-26 | 1.762316e-08 | LMM-Rank |
| PO_2_ | 0.0036055 | 2.852679e-09 | LMM-Rank |
| SO_2_ | 2.241866e-10 | 3.385285e-17 | LMM-Rank |
| Arterioles | | | |
| Diameter | 2.40068e-10 | 0.09854846 | LMM-Log |
| PO_2_ | 9.85794e-09 | 0.0004257559 | LMM-Rank |
| SO_2_ | 4.234369e-10 | 2.669722e-12 | LMM-Rank |
| Venules | | | |
| Diameter | 1.33486e-15 | 0.009041951 | LMM-Rank |
| PO_2_ | 7.822736e-06 | 1.544567e-12 | LMM-Rank |
| SO_2_ | 4.879744e-14 | 8.479095e-22 | LMM-Rank |
| OEF | | | |
| OEF artery-to-vein | 3.919964e-05 | 0.4806834 | LMM-Log |
| OEF arteriole-to-venule | 2.417526e-05 | 0.1340512 | LMM-Log |

LMM: Linear mixed model; LMM-Log: Linear mixed model with log transformed values (natural log); LMM-rank: Linear mixed model with ranked values; LM: Linear model; ANOVA: Analysis of variance; ANOVA-Log: Analysis of variance with log transformed values (natural log).

3) Test for normality of ELISAs

| Variable | Normality  Shapiro-Wilk test p-value | Log-normal  Shapiro-Wilk test p-value | Test selected |
| --- | --- | --- | --- |
| ICAM-1 - Cortex | 1.739592e-06 | 0.1088071 | ANOVA - Log |
| ICAM-1 - Hippocampus | 0.08856553 |  | ANOVA |
| CYP-A - Cortex | 0.004381789 | 0.02948101 | Kruskal-Wallis |
| CYP-A - Hippocampus | 0.6016157 |  | ANOVA |
| VEGFA | 1.33958e-05 | 5.913038e-05 | Kruskal-Wallis |
| Aβ-40 - Cortex | 0.4928973 |  | ANOVA |
| Aβ-40 - Hippocampus | 0.02649768 | 0.01783828 | ANOVA - Log |
| Aβ-42 - Cortex | 0.004118367 | 0.2288028 | ANOVA - Log |
| Aβ-42 - Hippocampus | 0.001796714 | 0.3593388 | ANOVA - Log |

LMM: Linear mixed model; LMM-Log: Linear mixed model with log transformed values (natural log); LMM-rank: Linear mixed model with ranked values; LM: Linear model; ANOVA: Analysis of variance; ANOVA-Log: Analysis of variance with log transformed values (natural log).
