# Supplementary material for: Carbonic anhydrase inhibitors prevent presymptomatic capillary flow disturbances in a model of cerebral amyloidosis
Source: Alzheimers Dement. 2025 Mar 25;21(3):e70023. doi: 10.1002/alz.70023 (PMC11936728; doi:10.1002/alz.70023)
Supplement: Supplementary file 3 — Supporting Information [file ALZ-21-e70023-s004.docx]

Carbonic anhydrase inhibitors prevent presymptomatic capillary flow disturbances in a model of cerebral amyloidosis

**Supplementary material 2**

**Supplementary figure 1** Visualization of MTT estimated by comparing AIF and VOF after time shifting the curves by the fitted MTT value (delay).

**Supplementary figure 2**. Examples of rejected curves. **A)** Example for bad fitting. Estimated venous output function (VOF) does not fit with recorded VOF. **B)** Example for signal saturation.

**Supplementary figure 3 | A)** Number of capillaries examined per ROI in each group. **B)** Stall incidence is not correlated with average cumulative stall duration per scanned region (LMM, p = 1). **C)** Probability distribution of the fraction of stalled segments and the cumulative duration. Tg+MTZ mice showed fewer capillary segments with large cumulative stalling duration (Kolmogorov-Smirnov test, p = 0.002).

**Supplementary figure 4**. **A)** Diameter coefficient of variance (COV) was estimated as the ratio of STD and diameter. WT vs. Tg-SwDI, LMM, diff = .0089 ± .0025, t(102) = 3.592, d = 0.995; Tg-SwDI vs. Tg+ATZ, LMM, diff. = – .00937 ± .00246, t(102) = –3.818, d = 1.041; Tg-SwDI vs. Tg+MTZ, diff. = – .01047± .00258, t(102) = –4.066d = 1.163. **B)** Vessel tortuosity was estimated as the segment length divided by the Euclidean distance between two segments ends. **C)** capillary blood volume (CapBV) was estimated as the percentage of volume occupied by the capillary segments in the total z-stack.

**Supplementary figure 5.** Regression plots of CTH as function of MTT from the mean estimated among all scanning trials (3 scans per mouse). Panels show the regression between CTH and MTT for **(A)** artery-to-vein (WT, n=20 scans in 8 mice; Tg-SwDI, n=21 in 13 mice; Tg+ATZ, n=20 in 10 mice; Tg+MTZ, n=29 in 12 mice) and **(B)** The linear model *cth∼mtt+factor(bolus),* which examines the relationship between CTH and MTT while controlling for the categorical effect of bolus. In the artery-to-vein network, no significant linear correlation is observed for the WT and Tg+MTZ groups, suggesting an active mechanism that regulates capillary flow to stabilize CTH across varying MTT levels. This indicates a compensatory response that maintains consistent oxygen extraction despite fluctuations in cerebral blood flow. In contrast, significant correlations observed in the Tg-SwDI and Tg+ATZ groups suggest a shift toward passive flow regulation, where CTH becomes increasingly dependent on MTT, potentially reflecting impaired vascular adaptability.

**Supplementary figure 6.** Arterial pO_2_ **(A)** and SO_2_ **(B)** as a function of vessel diameter. Linear regression was used to test if diameter predicted pO_2_and SO2 in the arterial network in all groups. We found that vessel diameter in the arterial network predicted pO_2_ (F(1,222) = 40.6, p < 0.001) and SO_2_ (F(1,222) = 38.4, p < 0.001) in the WT group. This prediction was not present in the Tg groups.

**Supplementary figure 7**. Subpopulation of plaques were done based on quantiles and classified as very small plaques (**A**), small plaques (**B)**; LMM, Tg-SwDI vs. Tg+ATZ rank-diff. = –16.9 ± 36.5, t(36.5) = –2.544, d = 1.101; Tg-SwDI vs. Tg+MTZ rank-diff. = –14.8 ± 6.42, t(35.6) = –2.298, d = .963), medium size plaques (**C**), and large plaques (**D**).

**Supplementary table 1.** Capillary stalling parameters across experimental groups

|  | WT  (n = 20 ROIs in 11 mice) | Tg-SwDI  (n = 34 ROIs in 18 mice) | Tg+ATZ  (n = 32 ROIs in 18 mice) | Tg+MTZ  (n = 30 ROIs in 15 mice) |
| --- | --- | --- | --- | --- |
| Incidence  (% of all capillaries) | 5.89 ± 3.37% | 8.42 ± 3.47% | 9.10 ± 3.34% | 7.92 ± 3.45% |
|  |  |  |  |  |
| Stalling events per OCT-A volume (Prevalence) | 0.39 ± 0.19% | 0.70 ± 0.43% | 0.63 ± 0.42% | 0.47 ± 0.28% |
|  |  |  |  |  |
| Cumulative time | 6.92 ± 2.52% | 8.38 ± 4.29% | 6.76 ± 3.56% | 5.89 ± 1.87% |
|  |  |  |  |  |
| Examined capillary segments | 160 ± 41 | 150 ± 35 | 158 ± 60 | 154 ± 29 |

**Supplementary table 2.** Capillary hemodynamics from single capillary line scans.

|  | WT  (n = 6 mice) | Tg-SwDI  (n = 7 mice) | Tg+ATZ  (n = 10 mice) | Tg+MTZ  (n =9 mice) |
| --- | --- | --- | --- | --- |
| Capillaries segments | 113 | 120 | 201 | 150 |
|  |  |  |  |  |
| Capillary diameter (µm) | 3.31 ± 0.45 | 3.21 ± 0.55 | 3.38 ± 0.52 | 3.64 ± 0.52 |
|  |  |  |  |  |
| RBCv (mm/s) | 0.98 ± 0.46 | 0.74 ± 0.36 | 1.00 ± 0.55 | 1.08 ± 0.58 |
|  |  |  |  |  |
| RBCv COV  (RBCv SD / RBCv mean) | 0.13 ± 0.12 | 0.12 ± 0.12 | 0.10 ± 0.07 | 0.10 ± 0.05 |
|  |  |  |  |  |
| Cell flux (cell/s) | 47 ± 17 | 42 ± 15 | 51 ± 21 | 56 ± 24 |
|  |  |  |  |  |
| Flux COV | 0.14 ± 0.07 | 0.16 ± 0.16 | 0.13 ± 0.07 | 0.15 ± 0.07 |
| (Flux SD / Flux mean) |  |  |  |  |
| LD (cells/mm) | 54 ± 18 | 63 ± 22 | 56 ± 19 | 58 ± 21 |
|  |  |  |  |  |
| LD COV  (LD SD / LD mean) | 0.14 ± 0.09 | 0.14 ± 0.10 | 0.13 ± 0.09 | 0.13 ± 0.08 |

**Supplementary table 3.** Capillary morphometrics from analysis of vascular stacks.

|  | WT  (n = 26 ROIs in 13 mice) | Tg-SwDI  (n = 25 ROIs in 13 mice) | Tg+ATZ  (n = 28 ROIs in 14 mice) | Tg+MTZ  (n = 23 ROIs in 12 mice) |
| --- | --- | --- | --- | --- |
| Capillaries segments | 4778 | 4566 | 4752 | 3676 |
|  |  |  |  |  |
| Mean Diameter per ROI (µm) | 5.67 ± 0.263 | 5.66 ± 0.36 | 5.67 ± 0.37 | 5.75 ± 0.41 |
|  |  |  |  |  |
| Mean STD per ROI (µm) | 1.179 ± 0.053 | 1.233 ± 0.08 | 1.182 ± 0.076 | 1.188 ± 0.051 |
|  |  |  |  |  |
| Mean CV per ROI | 0.22 ± 0.01 | 0.23 ± 0.01 | 0.22 ± 0.01 | 0.21 ± 0.01 |
|  |  |  |  |  |
| Capillary Tortuosity | 1.260 ± 0.026 | 1.276 ± 0.049 | 1.263 ± 0.052 | 1.268 ± 0.067 |
|  |  |  |  |  |
| Capillary Blood Volume (% of total z-stack volume) | 0.75 ± 0.24 | 0.63 ± 0.21 | 0.69 ± 0.26 | 0.61 ± 0.22 |
|  |  |  |  |  |
| Capillary Density (µm/µl) | 262385 ± 80468 | 220221 ± 76619 | 237403 ± 77891 | 203359 ± 71622 |

**Supplementary table 4.** MTT and CTH group values

|  | MTT | CTH |
| --- | --- | --- |
| S1: Artery to Vein |  |  |
| WT (n = 20) | 0.71 ± 0.18 | 0.44 ± 0.18 |
| Tg-SwDI (n = 21) | 0.70 ± 0.26 | 0.57 ± 0.26 |
| Tg+ATZ (n = 20) | 0.62 ± 0.19 | 0.52 ±0.19 |
| Tg+MTZ (n = 29) | 0.70 ± 0.22 | 0.54 ± 0.14 |
|  |  |  |
| S2: Arteriole to Venule |  |  |
| WT (n = 20) | 0.52 ± 0.22 | 0.52 ± 0.26 |
| Tg-SwDI(n = 18) | 0.50 ± 0.19 | 0.51 ± 0.17 |
| Tg+ATZ (n = 18) | 0.56 ± 0.17 | 0.67 ± 0.20 |
| Tg+MTZ (n = 26) | 0.47 ± 0.18 | 0.48 ± 0.11 |

**Supplementary table 5.** Pial vessel diameter in µm

|  | WT  (n = ) | Tg-SwDI  (n =) | Tg+ATZ  (n =) | Tg+MTZ  (n =) |
| --- | --- | --- | --- | --- |
|  |  |  |  |  |
| Arteries | 27.58 ± 9.83  *(n = 111)* | 30.69 ± 10.25  *(n = 101)* | 27.82 ± 9.29  *(n = 114)* | 28.25 ± 8.37  *(n = 129)* |
|  |  |  |  |  |
| Diving arterioles | 18.14 ± 5.35  *(n = 113)* | 18.17 ± 5.77  *(n = 106)* | 16.20 ± 3.36  *(n = 137)* | 18.61 ± 5.27  *(n = 145)* |
|  |  |  |  |  |
| Upstream venules | 19.35 ± 7.03  *(n = 172)* | 18.50 ± 6.94  *(n = 149)* | 16.96 ± 5.80  *(n = 163)* | 19.19 ± 6.66  *(n = 210)* |
|  |  |  |  |  |
| Veins | 40.32 ± 24.55  *(n = 99)* | 38.91 ± 19.18  *(n = 97)* | 41.10 ± 23.83  *(n = 88)* | 38.46 ± 19.63  *(n = 126)* |
|  |  |  |  |  |

**Supplementary table 6.** Mean PO2 (mmHg), SO2 (fractional) and OEF (fractional).

|  | WT  (n = ) | Tg-SwDI  (n =) | Tg+ATZ  (n =) | Tg+MTZ  (n =) |
| --- | --- | --- | --- | --- |
| **PO2** |  |  |  |  |
| Arteries | 87.4 ± 11.2  *(n = 111)* | 79.0 ± 14.7  *(n = 101)* | 77.4 ± 11.9  *(n = 114)* | 80.0 ± 13.2  *(n = 129)* |
|  |  |  |  |  |
| Diving arterioles | 82.5 ± 13.4  *(n = 113)* | 80.8 ± 19.2  *(n = 106)* | 74.5 ± 11.3  *(n = 137)* | 74.1 ± 15.7  *(n = 144)* |
|  |  |  |  |  |
| Ascending venules | 47.9 ± 9.1  *(n = 172)* | 44.5 ± 10.3  *(n = 149)* | 43.7 ± 8.7  *(n = 163)* | 49.2 ± 8.6  *(n = 210)* |
|  |  |  |  |  |
| Veins | 47.7 ± 8.2  *(n = 89)* | 48.4 ± 9.8  *(n = 91)* | 43.2 ± 9.8  *(n = 82)* | 48.9 ± 7.5  *(n = 116)* |
| **SO2** |  |  |  |  |
| Arteries | 0.87 ± 0.04  *(n = 111)* | 0.83 ± 0.07  *(n = 101)* | 0.83 ± 0.06  *(n = 113)* | 0.84 ± 0.05  *(n = 129)* |
|  |  |  |  |  |
| Diving arterioles | 0.85 ± 0.06  *(n = 113)* | 0.83 ± 0.09  *(n = 106)* | 0.82 ± 0.06  *(n = 137)* | 0.81 ± 0.08  *(n = 145)* |
|  |  |  |  |  |
| Ascending venules | 0.59 ± 0.12  *(n = 172)* | 0.54 ± 0.15  *(n = 149)* | 0.54 ± 0.13  *(n = 163)* | 0.61 ± 0.11  *(n = 210)* |
|  |  |  |  |  |
| Veins | 0.60 ± 0.11  *(n = 89)* | 0.60 ± 0.13  *(n = 91)* | 0.53 ± 0.15  *(n = 82)* | 0.61 ± 0.10  *(n = 116)* |
| **OEF** |  |  |  |  |
| Arteries to veins | 0.31 ± 0.11  *(n = 24)* | 0.30 ± 0.12  *(n = 25)* | 0.36 ± 0.15  *(n = 28)* | 0.28 ± 0.07  *(n = 34)* |
| Diving arterioles to ascending venules | 0.29 ± 0.11  *(n = 26)* | 0.33 ± 0.12  *(n = 25)* | 0.35 ± 0.14  *(n = 28)* | 0.24 ± 0.09  *(n = 34)* |

**Supplementary table 7. ELISA for amyloid load**

|  | WT  (n = ) | Tg-SwDI  (n =) | Tg+ATZ  (n =) | Tg+MTZ  (n =) |
| --- | --- | --- | --- | --- |
| Aβ-40 (pg/ml) - Cortex | 63 ± 61  (n = 12) | 85867 ± 19852  (n = 9) | 68450 ± 19852  (n = 9) | 73858 ± 20333  (n = 9) |
|  |  |  |  |  |
| Aβ-40 (pg/ml) - Hippocampus | 126 ± 161  (n = 9) | 75875 ± 29062  (n = 9) | 51824 ± 14892  (n = 9) | 73377 ± 26156  (n = 9) |
| Aβ-42 (pg/ml) – Cortex | 260 ± 242  (n = 12) | 1883 ± 668  (n = 9) | 1320 ± 280  (n = 9) | 1426 ± 233  (n = 9) |
|  |  |  |  |  |
| Aβ-42 (pg/ml) - Hippocampus | 338 ± 329  (n = 9) | 2289 ± 1158  (n = 9) | 1428 ± 230  (n = 9) | 1723 ± 364  (n = 9) |
