# Supplementary material for: Carbonic anhydrase inhibitors prevent presymptomatic capillary flow disturbances in a model of cerebral amyloidosis
Source: Alzheimers Dement. 2025 Mar 25;21(3):e70023. doi: 10.1002/alz.70023 (PMC11936728; doi:10.1002/alz.70023)
Supplement: Supplementary file 4 — Supporting Information [file ALZ-21-e70023-s001.docx]

Carbonic anhydrase inhibitors prevent presymptomatic capillary flow disturbances in a model of cerebral amyloidosis

**Supplementary Methods 1**

**Calculation of Expected Standard Deviation for Capillary Diameter Variability in treated and untreated Tg-SwDI mice**

For this calculation we assume that CAIs treatment prevented large variability resembling the WT groups.

To calculate the expected standard deviation (σ_adjusted_​), we first determined the capillary diameters at different distances from the pericyte soma for WT and Tg-SwDI groups. This was achieved by estimating the relative changes in capillary diameter between amyloid-associated and non-amyloid tissue, as reported by Nortley et al. (Figure 4D). Relative changes in diameter were calculated for distances of 4 µm, 7.5 µm, 12.5 µm, and 17.5 µm from the soma.

To align with the in vivo nature of our experiments and account for the differences between ex vivo and in vivo conditions, we avoided using standard deviation estimates directly from Nortley's study. Instead, we used the inner capillary diameter at the pericyte soma as a baseline measurement, obtained from WT and Tg-APP^NL−G−F^ mice in Korte et al. Using these in vivo baseline measurements, we calculated the expected diameters at each distance from the soma by applying the relative changes derived from Nortley et al.

The mean diameter ($\bar{D}$) for each group was then calculated as the average of all diameters across the distances considered. The variance (σ^2^) was computed by measuring the squared deviations of each diameter from the mean, normalized by the number of measurements. Given the assumption that pericytes influence capillary diameter over only 40 µm of the total 300 μm coverage as estimated by Hartmann et al., the variance was scaled by the proportion of the affected length:

$$Fraction Affected = \frac{40}{300} = 0.1333$$

The adjusted variance ($\sigma^{2}$​_adjusted_) was calculated by multiplying the original variance by this fraction:

$$\sigma_{\text{adjusted}}^{2}=\sigma^{2}\times\frac{40}{300}$$

Finally, the adjusted standard deviation was obtained as the square root of the adjusted variance:

$$\sigma_{\text{adjusted}}^{2}=\sqrt{\sigma_{\text{adjusted}}^{2}}$$

Assuming that treatment induced SD similar to WT (expected recovery), we estimated the SD for Tg-Ctrl and WT. The estimated standard deviations for WT and Tg-SwDI groups are summarized below:

| Group | SD (µm) |
| --- | --- |
| Tg-SwDI | 0.207 |
| WT (Expected recovery) | 0.182 |

The estimated relative change in SD between Tg-SwDI and treated mice is 13.73%.
